# Perovskite Electrocatalysts for Oxygen Evolution in Alkaline Media: From Fundamentals to Recent Developments

**DOI:** 10.1002/open.202500428

**Published:** 2026-03-26

**Authors:** Mikey Jones, Adeline Loh, Cheng Lyu, Ida Nawrocka, Jack Corbin, Zhenyu Zhang, Xiaohong Li

**Affiliations:** ^1^ Renewable Energy Group Department of Engineering Faculty of Environment Science and Economy University of Exeter Cornwall UK

**Keywords:** alkaline, electrocatalysis, green hydrogen, oxygen evolution reaction, perovskite

## Abstract

Carrying out electrolytic water splitting in an alkaline environment permits the usage of transition metal‐based electrode materials, compared to the significantly more expensive noble metal‐based materials required for electrolysis in an acidic environment. Of the two electrode processes that take place in a water electrolyser, the anodic oxygen evolution reaction (OER) is the bottleneck to the process, due to the sluggish kinetics of the reaction; thus, much research is being directed towards developing electrocatalysts to improve the efficiency of this process. Perovskites are a group of compounds that are emerging as promising OER electrocatalysts due to their low cost, good tunability and high catalytic activity. This review begins with the basic principles and fundamental limitations of the OER before exploring the trade‐offs between cost, activity, and durability that are often encountered in electrocatalyst design. Perovskites are then introduced as OER electrocatalysts, with a detailed discussion on structure, activity descriptors, catalyst design strategies, and synthesis methods. A critical review of recent advancements in perovskite materials as OER electrocatalysts is then presented at the end, including the latest developments in heteroatom doping and interface engineering.

## Graphical abstract

Perovskite oxides offer exceptional structural flexibility, enabling precise tuning of composition and electronic structure. This review connects these design parameters to both adsorbate‐ and lattice‐oxygen‐mediated OER pathways, highlighting the promise of rationally engineered perovskites as high‐performance electrocatalysts for alkaline water splitting.

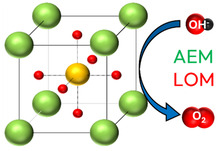

## 1 Introduction

Transitioning from a fossil fuel‐reliant energy network towards a system composed primarily of renewable energy sources will be one of the key challenges in mankind's efforts to reduce greenhouse gas emissions and environmental impact. In order to counteract the intermittency and unpredictability of most renewable energy sources, cheap, responsive, efficient and scalable energy storage solutions need to be developed and implemented extensively across all areas of the energy network. Hydrogen has long been championed as an ideal candidate for this task due to its high energy density and the fact that it only produces water when combusted in air or combined with O_2_ in a fuel cell [1, 2]. In addition to its credentials as an energy storage medium, hydrogen has a vital role across multiple industries, including transportation, food processing, steelmaking, and as an essential feedstock in the chemical industry for the manufacture of chemicals such as ammonia and sustainable aviation fuel [2].

In order to differentiate between the different hydrogen production processes, a colour palette is often utilised; amongst these, green hydrogen, produced via the electrolytic splitting of water molecules powered by renewable energy, can be classified as a truly sustainable and ‘clean’ production method, in contrast to fossil fuel‐based processes such as steam methane reforming and coal pyrolysis, which have colour designations ranging from turquoise through blue to black [3]. Unfortunately, more than 95% of the 7.94 Mt of hydrogen produced in Europe in 2023 came from a production pathway that is reliant on fossil fuels, mostly grey hydrogen from steam methane reforming [4], while the majority of hydrogen produced via electrolysis is generated as a by‐product in the chloro‐alkali sector (2%–3.5%) [5, 6]; in fact, in 2023 water electrolysis accounted for only 0.2% of total European hydrogen production and 0.1% of global dedicated hydrogen production [6, 7].

Alkaline water electrolysis (AWE) is by far the most deployed type of water electrolyser technology in the world today, accounting for 84% of global capacity deployed in 2023 [7]. AWE uses inexpensive and abundant materials such as first‐row transition metals (TMs), but suffers from low operating current density, poor efficiency, a large footprint, and relatively low gas purity. Proton exchange membrane water electrolysis (PEMWE) features a compact ‘zero‐gap’ design, enabled by the use of a proton exchange membrane as a solid polymer electrolyte (SPE) that electrically insulates the two electrodes in the cell. This zero‐gap design reduces the internal resistance of the system, allowing for higher efficiency operation at high current densities; it is more compact, allowing for higher capacity deployment in the same footprint; it improves product purity; and it has a fast response time, making it ideal for coupling to intermittent power sources such as solar and wind. There is, however, a drawback to this technology, as the harsh acidic operational conditions required for PEMWE necessitate that the electrode materials are composed of expensive and rare platinum group metals (PGMs) such as iridium, ruthenium and platinum, while titanium is generally used for components such as bipolar plates [2, 8]. It is thus highly desirable to develop an electrolyser that employs cheaper materials, as used in AWE, while utilising the zero‐gap design found in PEMWE, to enable the efficient, inexpensive production of green hydrogen. Anion exchange membrane water electrolysis (AEMWE) is one such technology, aiming to combine the benefits of both AWE and PEMWE technologies while negating their disadvantages. As renewable energy capacity increases and the cost of electricity trends downwards, electrolyser costs will become the driving force of the levelised cost of hydrogen, which needs to be reduced to <$1 USD / kg_H2_ by 2050, according to the International Energy Agency (IEA) [2]. This means that capital costs and maintenance costs will make up the majority of the future price of green hydrogen; this only further highlights the importance of developing low‐cost and efficient systems such as AEMWE.

**FIGURE 1:**
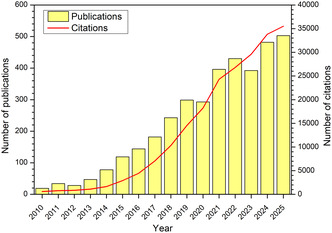
Number of publications and citations from 2010 to 2025 containing the keywords 'perovskite oxygen evolution'. All data was extracted from Web of Science.

The water splitting reaction is comprised of two separate half reactions: the hydrogen evolution reaction (HER) and the oxygen evolution reaction (OER), which take place at the cathode and anode of the electrolyser, respectively. The OER is considered to be the bottleneck to the reaction, due to the need to transfer twice as many electrons for each molecule of product gas evolved, compared to the HER. In order to reduce the potential required to drive the reaction, and thus improve the efficiency, it is necessary to develop and deploy anodic electrocatalysts. The use of alkaline conditions allows for the utilisation of cheaper TM‐based materials, compared with the PGM‐based materials that are required in an acidic environment. Hence, much research has been directed towards developing cheap, efficient, and durable materials for use as anodic electrocatalysts in alkaline environments, such as TM oxides [9, 10, 11, 12], hydroxides [13, 14, 15], oxyhydroxides [16, 17, 18], sulphides [19, 20], nitrides [21], and phosphates [22], amongst others. Of these materials, perovskites present a highly tuneable metal oxide structure that allows for a wide range of elements to be incorporated, thus offering an exciting route to making efficient catalyst materials; consequently, perovskite OER electrocatalysts have been the subject of intensifying research efforts for more than a decade. Figure 1 shows the increase of published articles accessible through the Web of Science interface across the years 2010–2025 (searched by the key words perovskite oxygen evolution). Besides their application as catalysts in low‐temperature water electrolysis, perovskite materials are also utilised widely in solid oxide fuel cells and electrolysers, photovoltaics, metal‐air batteries, ceramics, and non‐enzymatic sensors [23, 24, 25, 26, 27, 28].

There is a vast, ever‐growing body of work surrounding perovskite OER electrocatalysts. While several review articles on this topic already exist [29, 30], the growing interest in these materials calls for an up‐to‐date examination of the literature. The present review offers a distinct perspective by providing a comprehensive analysis of perovskite OER catalysts for alkaline water splitting, from the fundamentals of the OER through to some of the most recent developments in the field. The OER is first explored in detail, with the two main reaction mechanisms described, compared and evaluated, while fundamental limitations are discussed, before general OER performance indicators and challenges in catalyst design are covered, with suggestions on how to best design and assess catalysts based on the three main considerations of activity, durability, and cost. A detailed summary of the perovskite structure is then provided, along with OER activity descriptors, catalyst design strategies and perovskite synthesis procedures, before recent developments in perovskite OER catalysts are critically summarised, including multi‐metal doping, interface engineering, and the perovskite‐based Ruddlesden–Popper (RP) structures. We hope that this work will serve as a useful touchstone both to experienced perovskite electrocatalyst researchers and to those who are looking for a starting point in the topic.

## 2 The Oxygen Evolution Reaction (OER)

The OER is an anodic oxidation reaction and one of the two half‐reactions that constitute the overall electrolytic water splitting reaction, the other being the HER. The equilibrium half‐cell potentials (*E*_0_) at 1 atm and 298.15 K versus the standard hydrogen electrode (SHE) are shown below for OER (Equation (1)) and HER (Equation (2)) in alkaline conditions (pH = 14); the overall electrolytic splitting of water molecules proceeds via the reaction shown in Equation (3).

(1) $$4O{H^{‐}}_{\left(aq\right)}\to {O_{2}}_{\left(g\right)}+2H_{2}O_{\left(l\right)}+4e^{‐}\;\;\;\;\;\;\;\;E_{0}=+0.401V$$

(2) $$2H_{2}O_{\left(l\right)}+2e^{‐}\to H_{2\left(g\right)}+2{{\mathrm{OH}}^{‐}}_{\left(\mathrm{aq}\right)}\;\;\;\;\;\;\;\;E_{0}=-0.828V$$

(3) $$H_{2}O\to H_{2}+\frac{1}{2}O_{2}\;\;\;\;\;\;\;\;E_{0}=1.229V$$

Since the OER requires the transfer of four electrons for every molecule of O_2_ evolved, it is inherently sluggish when compared to the HER (which requires the transfer of two electrons for every molecule of H_2_ evolved), requiring a significant overpotential to drive the reaction. Here, the two typically accepted mechanisms for OER, based on the theories of intermediate adsorption and lattice‐oxygen evolution, are introduced.

### 2.1 Adsorbate Evolution Mechanism (AEM)

While the exact mechanism by which the OER proceeds has been the subject of much debate within the research community [31, 32, 33], it is generally agreed that it proceeds via four concerted proton‐electron transfer (CPET) steps on the surface sites of metal ions and involves the formation of several intermediate species [34]. The conventionally accepted steps are shown in Equations (4)–(7) [35]. In alkaline conditions, OH^−^ first adsorbs to the catalytic centre or adsorption site, depicted by M, to form an MOH intermediate. The MOH intermediate is then deprotonated to an MO intermediate and following this, another OH^−^ conducts a nucleophilic attack and adsorbs to the MO intermediate to form a MOOH intermediate. A final OH^−^ adsorbs to the MOOH intermediate, oxidising it and generating O_2_ on desorption.

Step 1:

(4) $$M+{\mathrm{OH}}^{-}\to \text{MOH}+e^{-}$$

Step 2:

(5) $$\mathrm{MOH}+{\mathrm{OH}}^{-}\to \text{MO}+H_{2}O+e^{-}$$

Step 3:

(6) $$\mathrm{MO}+{\mathrm{OH}}^{-}\to \text{MOOH}+e^{-}$$

**FIGURE 2:**
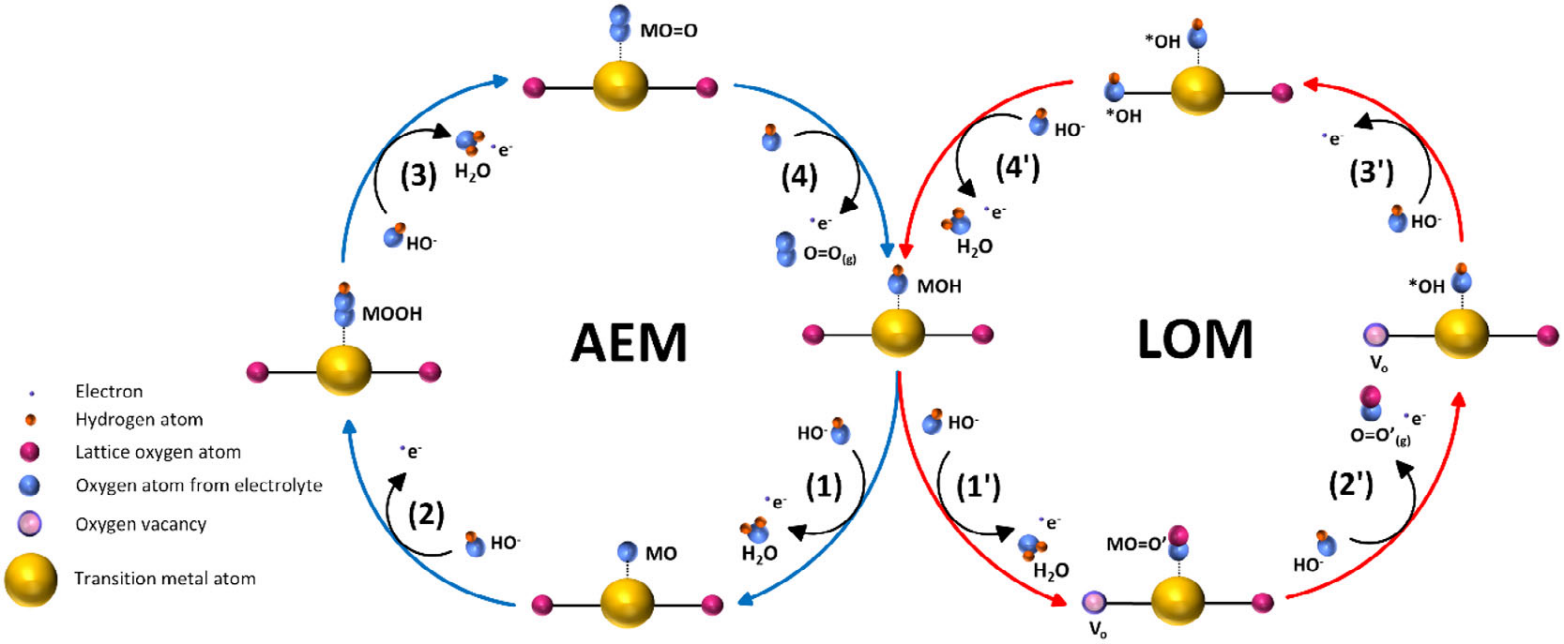
A diagram comparing the AEM and LOM routes on a representative perovskite oxide catalyst. Steps 1–4 correspond to Equations (4)–(7), steps 1′–4′ correspond to Equations (10)–(13).

Step 4:

(7) $$\mathrm{MOOH}+{\mathrm{OH}}^{-}\to M+O_{2}+H_{2}O+e^{-}$$

where M represents the metal catalytic site and MOH, MO and MOOH are the adsorbed intermediates. It is important to note that two of the MO intermediates formed after Equation (5) would be unable to recombine to form a molecule of O_2_ due to the large thermodynamic barriers that would be involved [36, 37]. This mechanism is known as the adsorbate evolution mechanism (AEM) and is depicted in Figure 2.

Electrocatalytic activity, and by extension overpotential, is largely governed by the binding strength of the oxygen‐containing intermediates to the catalyst surface according to the Sabatier principle [38]; the potential is limited by MOOH formation if surface‐oxygen binding is too strong and by MOH formation if surface‐oxygen binding is too weak [37, 39]. It is therefore preferable to find a catalyst that binds neither too weakly nor too strongly to the oxygen intermediates, a catalyst with a so‐called ‘goldilocks’ binding energy. The Gibbs free energy of each reaction step 1–4 (Equations (4)–(7)) provides a useful descriptor of the reaction kinetics and binding strength of the intermediate species [35]. The overall Gibbs free energy change for the OER (Δ*G*_OER_) is determined by the largest Δ*G* value, which is considered the rate‐determining step (Equation (8)). Generally, neither Δ*G*_1_ nor Δ*G*_4_ are considered to be rate‐limiting [37].

(8) $$\text{Δ}G^{\mathrm{OER}}=\max\left|{\text{Δ}G_{1}}^{0},{\text{Δ}G_{2}}^{0},{\text{Δ}G_{3}}^{0},{\text{Δ}G_{4}}^{0}\right|$$

**FIGURE 3:**
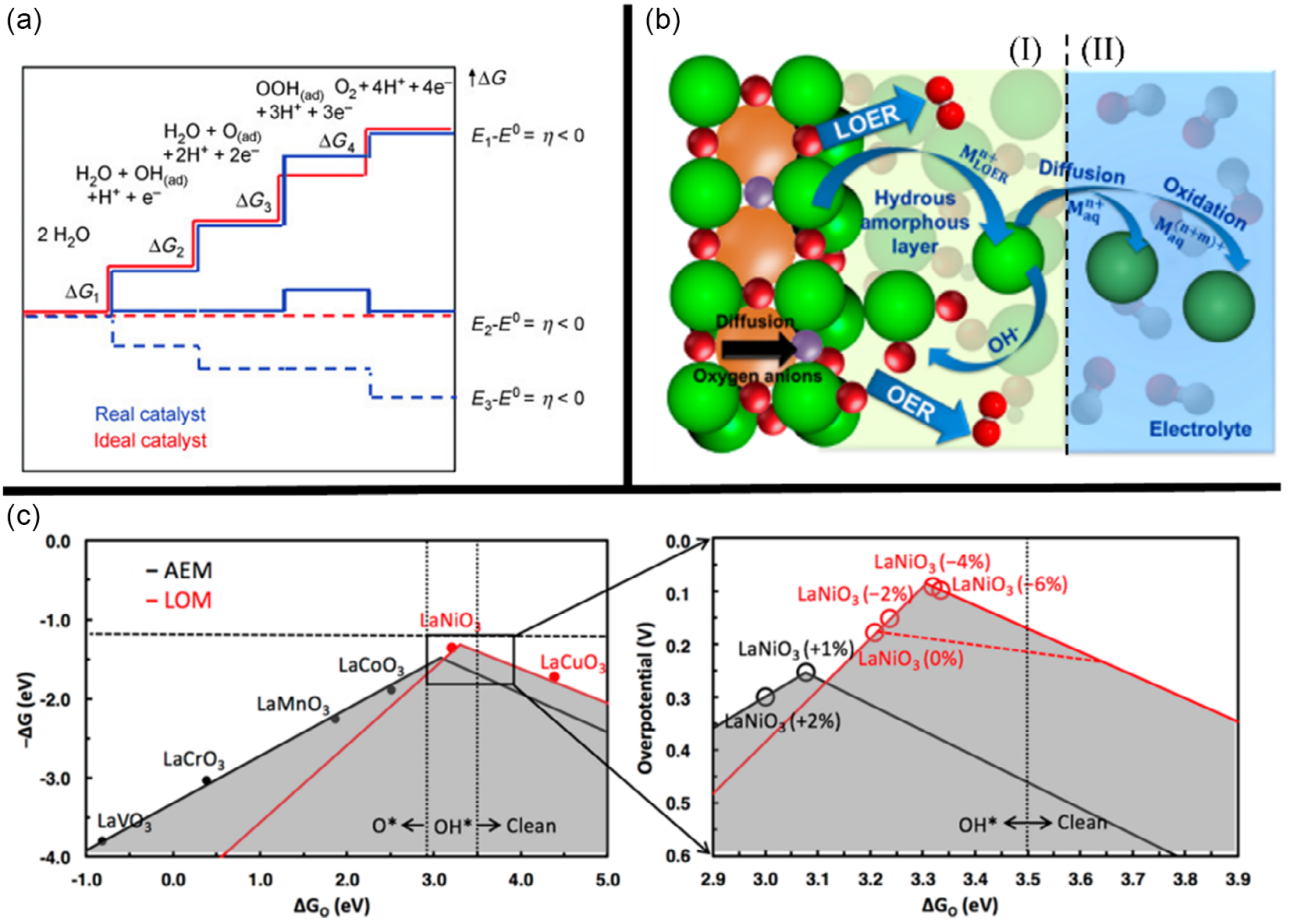
(a) Free energy diagram of the four‐step OER mechanism, reproduced with permission [40]. Copyright 2010, John Wiley and Sons; (b) OER/ LOM metal dissolution process initially proposed by Binninger et al. [41], figure adapted from [42], reproduced with permission Copyright 2018, American Chemical Society.; and (c) AEM and LOM participation of La‐based perovskites, reproduced with permission [43], Copyright 2018, American Chemical Society.

Man et al. found that the ideal OER catalyst would have reaction free energies of identical magnitude across each of the four steps at U = 0 (i.e., 1.229 eV) (Figure 3a), however, because the adsorption energies of the MO, MOH, and MOOH intermediates are linearly correlated this is near impossible to achieve in practice [44, 45, 46]. In particular, because both —OH and —OOH bind to the catalyst surface, M, via a single oxygen bond, their binding energies are closely linked and have, in fact, been found through density functional theory (DFT) studies to have a universal scaling relationship with an approximately constant difference of ≈3.2 eV [44, 46]. Since the two intermediates MOH and MOOH are separated by two CPET steps, the ideal separation energy is expected to be 2 times the standard cell potential, 1.23 eV, which equates to 2.46 eV. As the difference is greater than this, at 3.2 eV, a minimum theoretical overpotential of 0.37 V exists between an ideal and actual catalyst, which is calculated by Equation (9) [44, 45].

(9) $$\eta^{\mathrm{OER}}=\frac{\left(3.2\;\text{eV}-2.46\;\text{eV}\right)}{2e}=0.37\;V$$

The widespread accessibility of computational methods such as DFT has made predicting the activity of potential catalyst candidates a relatively common and useful practice. It should be noted that this theoretical thermodynamic overpotential often does not correlate with overpotentials found directly through laboratory experimentation. While the power and convenience of such in silico techniques are undeniable, it is important that the limitations of computer simulation are fully appreciated and that these methods are used to complement and support empirical data collected through rigorous laboratory work, rather than in lieu of such endeavours; a thorough discussion on the limitations of DFT is outside the scope of this text, but can be found in the following references [38, 47].

### 2.2 Lattice‐Oxygen Evolution Mechanism (LOM)

Since its discovery, the OER mechanism was for a long time understood solely based upon the binding strength of the reaction intermediates according to the Sabatier principle, and catalyst design has historically been guided by this concept [11, 12, 44, 48]. Although Bockris and Otagawa speculated about the involvement of lattice‐oxygen in the OER in the 1980s [33, 49], and indeed lattice‐oxygen participation of metal oxides had been observed through experimental studies in the early 21^st^ century [50], it was only really since the work of Binninger et al. in 2015 [41] that the idea of lattice‐oxygen participation in the OER started to gain traction within the research community. The initial work aimed to explain through thermodynamic reasoning the experimentally observed corrosive phenomena that were directly correlated with the onset of OER on metal oxides; namely, the dissolution of metal cations into the electrolyte and the appearance of lattice‐oxygen in the evolved O_2_ gas [51]. The authors successfully derived the impossibility of metal oxide stability under OER conditions, showing that the applied overpotential for the lattice oxygen mechanism (LOM) is always greater than the overpotential for OER, at a fixed electrode potential; a derivation that has since been made more succinct by Exner [52]. Binninger et al. found that the LOM could be energetically preferential to the conventional OER mechanism, as seen in Figure 3c), while in alkaline conditions the AEM and LOM are generally kinetically and thermodynamically coupled [41].

In the LOM, the metal cations from the bulk metal oxide are either oxidised to a higher valence state during solvation in the electrolyte (part II in Figure 3b) or react with OH^−^ in the electrolyte to form a hydrous amorphous layer (part I in Figure 3b). Fabbri et al. have suggested that the formation of this hydrous layer can enhance OER performance by stabilising the process [42, 53]. This occurs through the recombination of metal cations that result from the LOM with OH^−^ from the electrolyte, allowing the evolved lattice oxygen ions to be continually replaced by the OH^−^ in the electrolyte, rather than the dissolution and oxidation of the metal cations to a higher valence state, which would eventually result in degradation of the electrode material due to loss of mass.

Equations (10)–(13) give the most widely proposed mechanism for the LOM [54, 55], where M is the B‐site metal, O′ refers to lattice oxygen and V_o_ refers to a surface lattice oxygen vacancy. In the LOM, lattice oxygens act as active sites leading to the reversible formation of oxygen vacancies, which can then be filled by OH^−^ ions from dissociated water or OH^−^ ions from the electrolyte.

Step 1′:

(10) $$\begin{array}{c} \mathrm{OH}\\ |\\ O^{\prime }-M-O^{\prime } \end{array}+{\mathrm{OH}}^{-}\to \begin{array}{c} {\mathrm{OO}}^{\prime }\\ |\\ O^{\prime }-M-V_{o} \end{array}+H_{2}O+e^{-}$$

Step 2′:

(11) $$\begin{array}{c} {\mathrm{OO}}^{\prime }\\ |\\ O^{\prime }-M-V_{o} \end{array}+{\mathrm{OH}}^{-}\to \begin{array}{c} \mathrm{OH}\\ |\\ O^{\prime }-M-V_{o} \end{array}+O_{2}+e^{-}$$

Step 3′:

(12) $$\begin{array}{c} \mathrm{OH}\\ |\\ O^{\prime }-M-V_{o} \end{array}+{\mathrm{OH}}^{-}\to \begin{array}{c} \mathrm{OH}\\ |\\ O^{\prime }-M-\mathrm{OH} \end{array}+e^{-}$$

Step 4′:

(13) $$\begin{array}{c} \mathrm{OH}\\ |\\ O^{\prime }-M-\mathrm{OH} \end{array}+{\mathrm{OH}}^{-}\to \begin{array}{c} \mathrm{OH}\\ |\\ O^{\prime }-M-O^{\prime } \end{array}+H_{2}O+e^{-}$$

Unlike the AEM, where the CPET means that deprotonation and electron transfer occur at the same time, the LOM is reliant on a non‐concerted proton and electron transfer (nCPET), where the adsorbed OH^−^ group is first deprotonated and subsequently an electron is transferred from O^2−^ to the external circuit [56]. While the electron transfer is determined by the redox potential of the active site and the applied potential, the proton transfer is dependent on the acid dissociation constant (*K*_a_) for the deprotonation of OH^−^, meaning that the transfer of the proton is limited by the pH value of the electrolyte and thus the LOM displays pH dependence [56, 57, 58]. It is widely considered that catalysts operating via the LOM present the opportunity to bypass the theoretical minimum overpotential of the AEM [43, 59, 60] there is still much debate over what effect, if any, this has on catalyst stability [52, 61]. Furthermore, there are arguments that LOM may be undesirable from a charge efficiency point of view, as a correlation has been found between catalysts with higher LOM participation suffering from lower Faradaic efficiency of O_2_ [62].

## 3 Evaluating Catalyst Materials

### 3.1 The Ideal OER Catalyst

**FIGURE 4:**
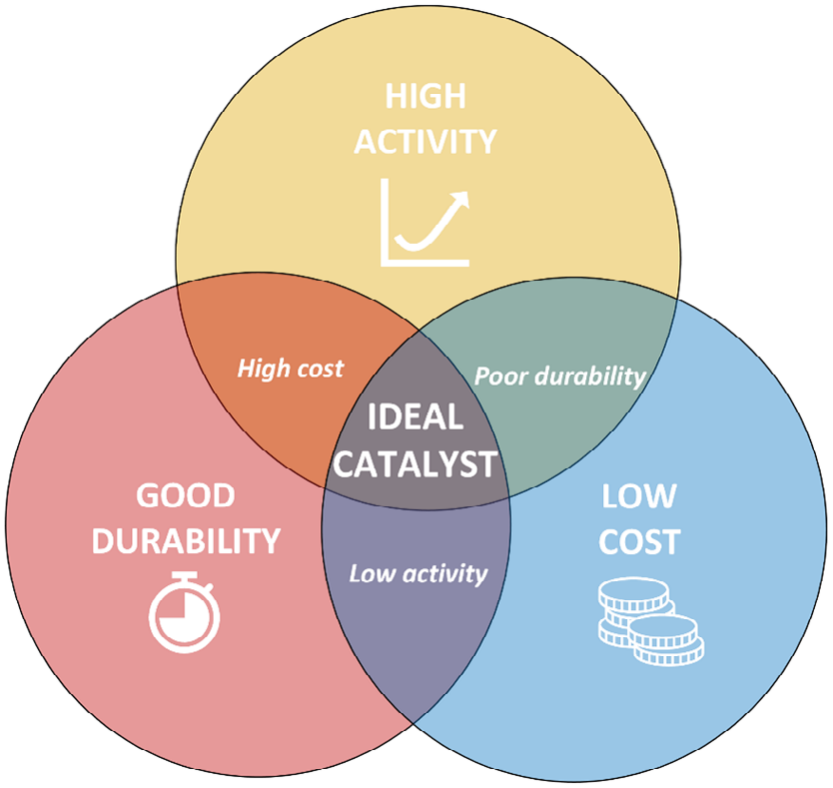
Venn diagram illustrating the common trade‐offs between catalyst activity, durability, and cost.

The ideal catalyst for the electrochemical evolution of oxygen should possess exceptional intrinsic activity, a large active surface area, excellent electrical conductivity, and outstanding long‐term stability and durability under industrially relevant conditions, such as elevated temperature with highly concentrated electrolyte. Additionally, it should be easily manufactured at scale for a low cost from inexpensive, earth abundant and readily available materials. To this end, many different avenues of research are currently being explored in pursuit of the ideal OER electrocatalyst, including investigations into the fundamentals of the OER mechanism [49, 54, 63, 64, 65], examination and analysis of various groups of catalysts and substrates [10, 66, 67, 68, 69, 70], development of different methods for tuning the performance and durability of catalysts [71, 72, 73, 74], and evaluation of the most suitable catalyst synthesis and electrode preparation methods [75, 76]. As a material that fulfils every criterion is yet to be found, trade‐offs between activity, durability and cost often have to be made, as shown in Figure 4.

First‐row TM‐based catalysts offer a good compromise between cost and performance. Although they are generally less catalytically active than PGMs, their significantly lower cost and good stability in alkaline media make them attractive candidates for catalyst development. In particular, the oxides and hydroxides of both Ni and Co exhibit excellent OER activity, which can be enhanced further by the presence of a small amount of Fe dopant [11, 12, 77, 78, 79, 80, 81, 82]. Whilst carrying out water splitting in alkaline environments allows for the use of TM‐based catalysts, PGMs must be used in acidic media because most other metals are susceptible to oxidative corrosion. Nevertheless, the best performing OER catalysts in acidic environments are also the benchmark for alkaline media; these are IrO_2_ and RuO_2_, which both exhibit high catalytic activity resulting in low overpotentials. However, the real‐world application of these catalysts is limited by their very high cost and scarcity, and, in the case of RuO_2_, insufficient long‐term stability under OER conditions further limits its viability [83]. Regardless, these catalysts serve as an excellent point of reference to measure the performance of other OER catalysts.

### 3.2 Assessing Catalyst Materials

It is necessary to be able to make direct comparisons between different catalysts to assess the three most important criteria: catalytic activity, durability, and cost. Herein, we describe the most commonly used metrics and techniques for assessing these attributes.

#### 3.2.1 Catalytic Activity

The conventionally reported electrocatalysis activity markers are overpotential (*η*_*j*_) at selected current densities *j*, where *j* is most often equal to 10 mA cm^−2^; Tafel slope; and exchange current density. While the overpotential at a current density of 10 mA cm^−2^ (η_10_) is by far the most reported performance metric in OER electrocatalysis studies, it is imperative that performance is also reported at higher current densities that are more relevant to real‐world applications in industrial settings; reporting values for η_100_, η_500_, and, if possible, ≥η_1000_ allows for easy comparison between high‐performance electrocatalyst materials and enables quick assessment of their viability as candidates for further development; to date, the best performing OER catalysts report η_1000_ values below 300 mV and η_2000_ values below 450 mV [84, 85, 86]. Catalysts that exhibit a low overpotential at high current densities enable more efficient catalytic reactions: the lower the overpotential, the more efficient the process. While ultimately the performance goal for any electrocatalyst should be to rival the activity of the best performing materials, given the relative immaturity of research into perovskite OER electrocatalysts we suggest a η_500_ value of below 300 mV as a reasonable screening target to identify promising catalyst materials for future development, as 500 mA cm^−2^ is the realistic minimum current density that a perovskite electrocatalyst will be required to operate at and the 300 mV threshold has already been achieved by many TM‐based catalysts and a few state‐of‐the‐art perovskite materials [13, 87, 88]; we emphasise that this target should be regarded as a pragmatic screening benchmark rather than an ultimate performance limit.

To achieve this, a catalyst must be highly active towards the OER process. One strategy for achieving this is to increase the surface area of the catalyst, thus increasing the number of accessible active sites. Due to the large differences in reported catalyst surface areas, it is good practice to report both overpotential at a current density corresponding to the geometric area of the electrode and current density normalised to the electrochemical surface area (ECSA) of the material, particularly when porous materials such as foams are utilised as a catalyst substrate. ECSA can be calculated in a number of ways, the most widely utilised approach being the double layer capacitance (*C*_dl_) method, where the gradient is taken from a plot of the non‐Faradaic capacitive current (*i*_c_) as a function of different cyclic voltammogram (CV) scan rates (υ) and divided by the specific capacitance (*C*_s_) of the material, as the charging current of a capacitor is equal to the capacitance multiplied by the change in voltage with respect to time. The value of *C*_s_ will vary depending on the material, and finding a reliable value in the literature can be challenging; many researchers therefore opt to use a general value of 0.040 mF cm^−2^ when dealing with alkaline systems [89].

Tafel analysis is another important and widely used method for reporting catalyst activity and can provide information on the rate‐determining step of a reaction mechanism. Defined as the potential required to increase the current by a factor of 10, with units of mV dec^−1^, reported values for OER Tafel slopes are usually between 30 and 120 mV dec^−1^, with a lower Tafel slope generally indicating more favourable reaction kinetics [90, 91]. In order to obtain an accurate Tafel slope value, it is essential that there are no capacitive currents, meaning that either linear sweep voltammetry (LSV) with a very low scan rate (≤1 mV s^−1^) should be used to simulate steady‐state conditions or, more ideally, chronoamperometry (CA) with a long enough hold time at each potential step for the system to reach a steady state. Extrapolating the plotted Tafel slope to *η *= 0 mV gives the exchange current density *j*_0_, which is a measure of the charge transfer rate at the equilibrium potential and reflects the intrinsic activity of the catalyst; a high *j*_0_ value is desirable in electrocatalysis, as this indicates that there is extensive oxidation and reduction occurring at the equilibrium potential, meaning that a smaller activation overpotential is needed to deliver a net current than for a system with a smaller exchange current density [92, 93].

Onset potential is often reported as an activity marker for electrocatalysis, this parameter is poorly defined in the literature. Researchers often state a specific current density as the onset point of the reaction, however this approach can be problematic as it can be difficult to distinguish between Faradaic and non‐Faradaic processes, and the reported onset point can vary widely from as little as 10 µA cm^−2^ to as much as 10 mA cm^−2^, making it difficult to draw comparisons between different studies; it is therefore argued that reporting the specific‐activity or mass‐activity (A g^−1^) of a material is a more valuable metric for catalytic activity [94].

Faradaic efficiency is also a useful measure of catalyst performance and describes how much of the transferred charge is converted into the desired product; however, in order to calculate this, it is necessary to quantify the amount of product gases created, requiring either offline or online analysis via techniques such as gas chromatography (GC) or differential electrochemical mass spectrometry (DEMS).

#### 3.2.2 Durability

**FIGURE 5:**
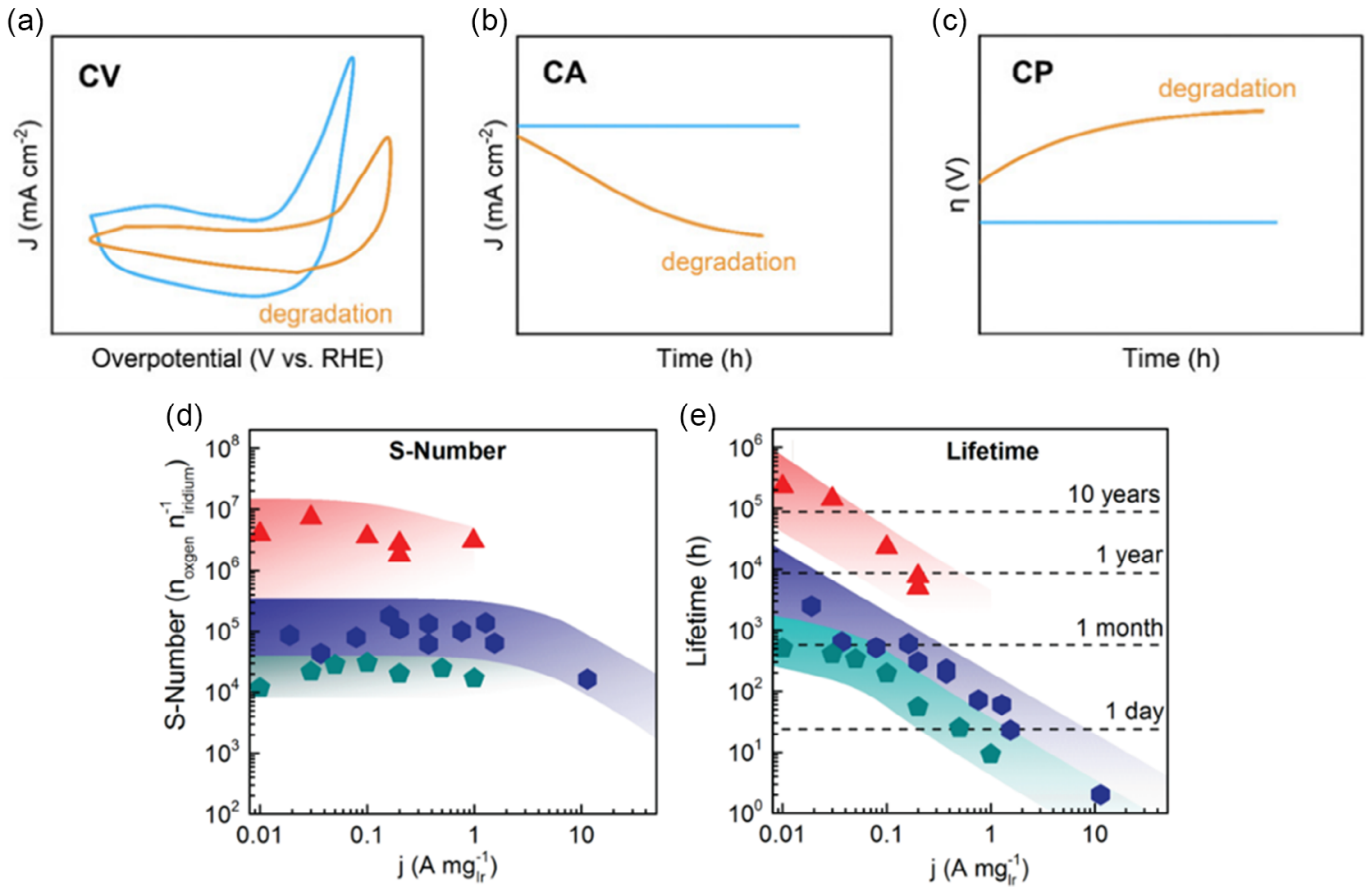
(a–c) Stability assessment using(a) cyclic voltammetry, (b) chronoamperometry, and (c) chronopotentiometry, reproduced with permission [95], Copyright 2024, Royal Society of Chemistry; (d,e) investigation of S‐Number and lifetime of various Ir oxides depending on the current load; (d) stability‐number (S‐Number) plotted versus mass specific current density and (e) calculation of the catalyst's lifetime, based on (15, reproduced with permission [96], Copyright 2018, Springer Nature.

The stability and durability of electrocatalysts are generally assessed through a combination of three different electrochemical techniques: CV, CA, and chronopotentiometry (CP) (Figure 5a–c). In CV, the potential is continuously scanned at a chosen scan rate over a defined potential range, and the current response is measured. In general, the smaller the change in the current response over multiple cycles, the greater the stability of the material. CA measures the change in current density with respect to time at a set potential, while CP measures the change in potential with respect to time at a fixed current density. As CA is carried out at constant potential, this means that the electro‐oxidation conditions are constant, whereas in CP the changing potential indicates a change in electro‐oxidation conditions, which can result in inconsistencies between the measurements; as such, a combination of the abovementioned methods should be used to obtain reliable stability information [95]. Commercial electrolysers have a lifetime in excess of 50,000 h [2], meaning that it is essential that any water splitting electrocatalyst has excellent stability and undergoes minimal degradation over the lifetime of the electrolyser, keeping the OPEX, and thus the price of green H_2_, as low as possible. Current state‐of‐the‐art AWE stacks have degradation rates as low as 3 µV/h [97], while the U.S. Department of Energy's ultimate target AWE and PEM stack degradation rate is 2 µV/h [98], which corresponds to ~10% voltage loss over 80,000 h of operation. Taking this into account, we suggest that a degradation rate of 8 µV/h is a useful criterion for materials discovery; this deliberately conservative target, corresponding to ~20% voltage loss over 50,000 h, is meant to filter out compositions that exhibit rapid intrinsic degradation on laboratory timescales, ensuring that only candidates with at least quasi‐stable behaviour under benchmark conditions are advanced for more detailed study.

However, it is not feasible to test materials over such long time periods; as such, accelerated stress testing is an important tool that is used to simulate the long‐term operation of electrodes over a shorter testing duration and ascertain whether a catalyst has promising stability in a relatively short time frame [99, 100]. For example, CV cycling over many thousands of cycles at a high scan rate can result in catalyst dissolution, passivation, and delamination and is therefore deemed to be a useful way of quickly learning about a material's stability [80]. Accelerated stress testing regimens should be coupled with ex situ investigations such as scanning electron microscopy (SEM), X‐ray photoelectron spectroscopy (XPS), and inductively coupled plasma mass spectrometry (ICP‐MS) to learn more about degradation mechanisms such as particle detachment and cation dissolution. Geiger et al. propose a metric, dubbed the stability number (S), that characterises the activity versus stability performance of a given catalyst, defined as the ratio between the amount of evolved gaseous product, calculated from total charge passed, and the amount of electrocatalyst active site cations dissolved in the electrolyte, found through ICP‐MS, (Equation (14)); the higher the number, the more stable the active centre of the catalyst [96]. From this S‐number, the lifetime of the catalyst can be evaluated (Figure 5) using Equation (15), with the assumption that Faradaic efficiency is 100% and dissolution proceeds at a constant, steady state, while neglecting an increase in dissolution towards the end of life due to a loss of surface area.

(14) $$S=\frac{{\mathrm{nO}}_{2}}{{\mathrm{nM}}_{\text{dissolved}}^{z+}}$$

where *S* = stability number, nO_2_ is the moles of evolved oxygen gas, and ${\mathrm{nM}}_{\text{dissolved}}^{z+}$ is the moles of cation M^z+^ dissolved in the electrolyte.

(15) $$t=\frac{S\cdot z\cdot F\cdot m}{j\cdot M}$$

where *t* = catalyst lifetime (s), *S* = stability number, *z* = number of electrons transferred per O_2_, *F* = Faraday constant, *m* = loaded mass of metal (g cm^−2^), *j* = applied current density (A cm^−2^), *M* = molar mass of metal.

While the ability to estimate catalyst lifetime after only a short amount of testing is undoubtedly advantageous, this proposed metric is yet to see widespread adoption within the field, possibly because of the necessity for specialised ICP‐MS analysis, which is often not easily accessible to many research groups. It is important to also note that the S‐number only accounts for the physical degradation of the catalyst and does not consider degradation of catalysts by other routes, such as chemical deactivation of catalytic sites. The adhesion of the catalyst layer to the electrode substrate is of utmost importance, particularly when carrying out longer‐term durability testing of catalyst materials, as it is important to be able to distinguish between phenomena such as the chemical deactivation of catalytic sites and the physical detachment of catalysts from the electrode surface, both of which will be observed as a decrease in electrochemical performance during testing. Catalysts are often synthesised as powders, and as such need to be deposited onto a substrate such as glassy carbon or nickel foam in order to carry out electrochemical testing. Catalyst powders produced via methods such as sol–gel synthesis can be made up into ink and coated onto the substrate through methods such as spray coating, drop‐casting or dip‐coating [101]. Commonly, ionomers are added to the ink solution before deposition to achieve good catalyst adhesion to the substrate and to facilitate good ionic conduction [102]. Conductive additives such as carbon black can also be added to improve the electronic conductivity of the catalyst layer [103, 104]. Any such additions should be carefully considered, as both can have a negative effect on the stability and performance of the electrode. Ionomer content affects both the mechanical stability and activity of the catalyst layer; adding too much ionomer can result in the blocking of catalyst active sites, while using too little can lead to catalyst detachment. Meanwhile, the instability of carbon at highly anodic potentials renders it unsuitable for long‐term applications; nevertheless, carbon black is the most commonly added conductive support and Filimonenkov et al. report a protective effect from highly active OER catalysts that minimises carbon corrosion, making it suitable for catalyst screening purposes, generally in a traditional three‐electrode configuration [103, 104]. Longer duration testing is best carried out in a full‐cell configuration using a membrane electrode assembly (MEA), where the catalyst of interest is coated onto either a conductive substrate such as nickel foam (known as catalyst coated substrate, CCS) or onto the SPE (known as catalyst coated membrane, CCM). Some of the most commonly used SPEs for AEMWE include the Fumasep FAA‐3 by Fumatech, Aemion by Ionomr, and Sustainion by Dioxide Materials [105].

#### 3.2.3 Cost

**FIGURE 6:**
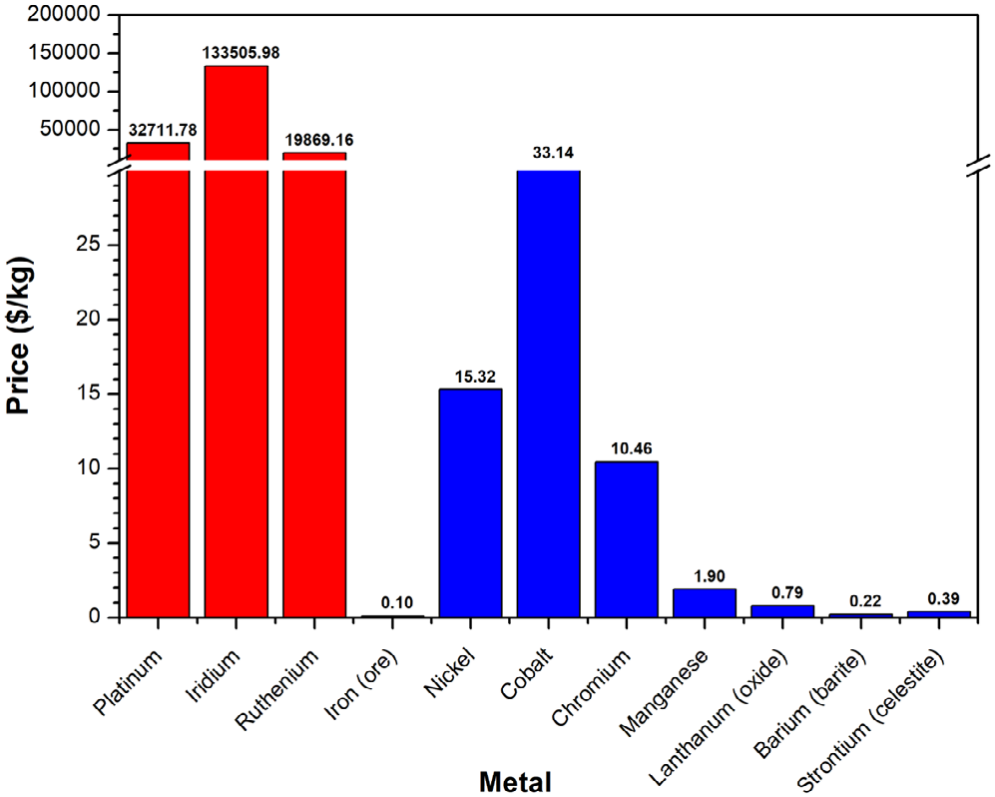
Price (US$/kg) of various metals (and their precursors) commonly used as electrocatalysts in water electrolysis; PGMs are red, TMs are blue. Correct as of May 2025 [106, 107].

There are two primary cost considerations that should be considered when designing catalyst materials: the cost of raw materials and the cost of manufacturing electrocatalysts from these raw materials. Ideally, the material manufacturing process should be easily scalable, cost effective, and have a high yield with a low energy consumption. The financial cost of PGM materials is one of the primary cost drivers of PEMWE systems; comparatively, the TM‐based materials used in AWE and AEMWE are much cheaper than PGMs (Figure 6). However, many of the non‐PGM elements commonly used for the manufacture of electrode materials have been designated as critical raw materials (CRMs) and strategic raw materials (SRMs) by the EU, such as Ni and Co [108]. While it is easy to take the cost of materials at face value, it is also important to consider future demand for these materials from other sectors. For example, both Co and Ni are used extensively in Li‐ion batteries, and clean energy technologies’ share of total demand for these two metals is expected to increase to 60%–70% in the next two decades, while EVs and battery storage in particular are forecast to take over from the stainless‐steel industry as the largest end user of Ni by 2040 [109]. This substantial increase in demand could cause supply issues in the future, resulting in a spike in the price of the materials. While the financial cost of materials is going to be the main driver of the price of green H_2_, it is also important to consider the environmental and social costs of certain materials when designing electrocatalysts. Minerals containing metals such as Co often come from ethically ambiguous sources, where the extraction of raw materials and wealth is prioritised over human rights and with little regard for the impact on the natural environment [110]. As such, life cycle analysis is an important tool that should be utilised alongside the design of catalytic materials, in order to limit the extraction of these materials and encourage reuse and recycling as much as possible. It is important that all materials are responsibly sourced, to ensure that indigenous populations are able to benefit from the natural resources in their homelands, rather than be exploited, while preserving delicate ecosystems as much as possible. To this end, working to minimise the CRM and SRM content of electrocatalysts while considering the future recyclability of the materials is an important secondary consideration when designing catalyst materials.

## 4 Perovskite OER Electrocatalysts

The perovskite family of compounds take their name from the mineral perovskite and shares the same basic formula, ABX_3_, where A is a cation of charge 2+ or 3+, B is a cation of charge 3+ or 4+, and X is an anion such as oxygen or a halide. First described by Gustav Rose in 1839 from a rock sample collected in the Ural Mountains and named after the Russian aristocrat and mineral collector Count Lev Alekseevich von Perovskiy [111], the chemical composition of the mineral perovskite is CaTiO_3_, meaning that it technically falls into the perovskite oxide sub‐group of the wider perovskite family. Furthermore, while the ideal perovskite crystal structure is cubic, CaTiO_3_ belongs to the orthorhombic crystal system [112]. These inconsistencies have led to some advocating for a renaming of the broader structural family over the years [113], however the perovskite name has endured. Today, compounds are described as having a perovskite‐type structure. For the overall ionic structure of the perovskite to be neutral, it must fulfil (Equation (16)**)**:

(16) $$q_{A}+q_{B}=-3q_{X}$$

where *q*_A_ is the charge on cation A, *q*_B_ is the charge on cation B and *q* *_X_* is the charge on anion X [114]. The ability to substitute elements with varying electronegativity, valency and ionic size into the A, B, and X‐sites provides the useful ability to alter and tune the electronic, physical, and structural properties of the compound [115].

### 4.1 Perovskite Crystal Structure

**FIGURE 7:**
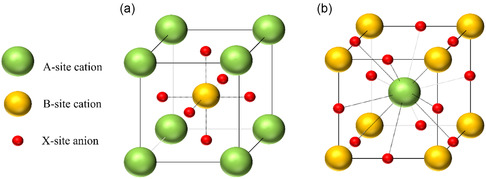
The perovskite ABX_3_ lattice with (a) the A‐site cation as the origin atom and (b) the B‐site cation as the origin atom. The A‐site cation adopts a 12‐fold coordination of the X‐anion while the B‐site cation is octahedral, with a sixfold coordination of the X‐anion.

The ideal, aristotypical perovskite structure has cubic symmetry, with the space group *Pm* $\overset{―}{3}$ *m*; the A‐site cations are situated at cube corner positions (0, 0, 0), the B‐site cations are located at the body centre positions (1/2, 1/2, 1/2) and the X‐site anions sit at face‐centred positions (1/2, 1/2, 0), (1/2, 0, 1/2) and (0, 1/2, 1/2) (Figure 7a) [116]; alternatively, the A‐site cations can be depicted in the body centre positions with the B‐site cations in the cube corner positions with the X‐site anions sitting at (0, 0, 1/2), (0, 1/2, 0), and (1/2, 0, 0) positions (Figure 7b) [114].

Typified by SrTiO_3_ at room temperature, the ideal perovskite structure is cubic close‐packed, with the Sr and O atoms stacked along the cubic [1 1 1] direction in cubic close‐packed layers, and Ti atoms occupying some of the resulting octahedral holes. The TiO_6_ octahedra are geometrically ideal, with 90° bond angles and six equal Ti—O bonds of length 1.952 Å; meanwhile, each Sr atom is surrounded by twelve oxygen atoms, each equidistant at 2.761 Å [117]. In 1926, the pioneering work of Goldschmidt developed an equation that used the radius ratios between ions to describe the different structures of coordination compounds [118]. This equation resulted in the tolerance factor, *t*, describing how far from ideal the packing in the structure can be whilst still holding the perovskite structure; in other words, the tolerance factor describes the extent of distortion in the perovskite unit cell structure.

(17) $$t=\frac{r_{A}+r_{X}}{\sqrt{2}\left(r_{B}+r_{X}\right)}$$

where *r*_A_, *r*_B_, and *r*_*X*_ represent the ionic radii of the A‐site, B‐site, and X‐site ion, respectively; values are often taken from Shannon's table [119, 120]. It was initially posited that a tolerance factor between 0.8 and 1.0 would result in a stable perovskite‐type structure; however, stable perovskites have since been reported with tolerance factors between 0.71 and 1.1 [121, 122]. When *t* ≈ 1.0, the crystal structure presents as the ideal cubic structure; deviations from this ideal value lead to hexagonal, tetragonal, rhombohedral, orthorhombic, and other structures, with the specific configuration depending on the value of *t* [24]. When *t* = 0.9–1.0 the structure exhibited is likely cubic, with both A‐ and B‐site cations possessing ideal size [114]. At values of *t* = 0.71–0.9 leads to crystal structures with a lower symmetry than cubic, usually either an orthorhombic, rhombohedral, or tetragonal structure, due to the A cations being too small to fit into B‐site interstices, which can result in elongation of the unit cell along one or more directions and/or distortions in the central BX_6_ octahedra, most commonly expressed as tilting and rotation about one or more of its three axes [121]. When *t* < 0.71, other structures such as trigonal or tetragonal can present, due to the A and B cations being of similar size [114, 121]. If *t* > 1, then the A cation is larger than ideal while the B cation is smaller, leading to hexagonal‐close‐packed stacking of the AX_3_ layers with face‐sharing octahedra, which results in shorter metal–metal distances and reduced metal–oxygen–metal bond angles compared to perovskites with a more ideal structure [122, 123]. Doping perovskite materials with elements of differing ionic radii affects the crystal structure and subsequently the electronic structure of the material and is therefore an important approach used for designing catalyst materials, which will be detailed in Section 4.3.

### 4.2 Perovskite OER Activity Descriptors

Researchers have sought to identify descriptors for the OER activity of perovskite materials to best inform catalyst design approaches. However, this has proven to be difficult due to the fact that altering a single property, such as the oxygen vacancy content, also modifies properties such as the conductivity, crystal structure, electronic configuration and cation ordering within the material [124]. Nevertheless, two separate activity descriptors have been proposed and are generally accepted within the field: *e*_g_ orbital occupancy and the covalency of the bond between the B‐site metal and lattice oxygen.

#### 4.2.1 e_g_ Orbital Occupancy

**FIGURE 8:**
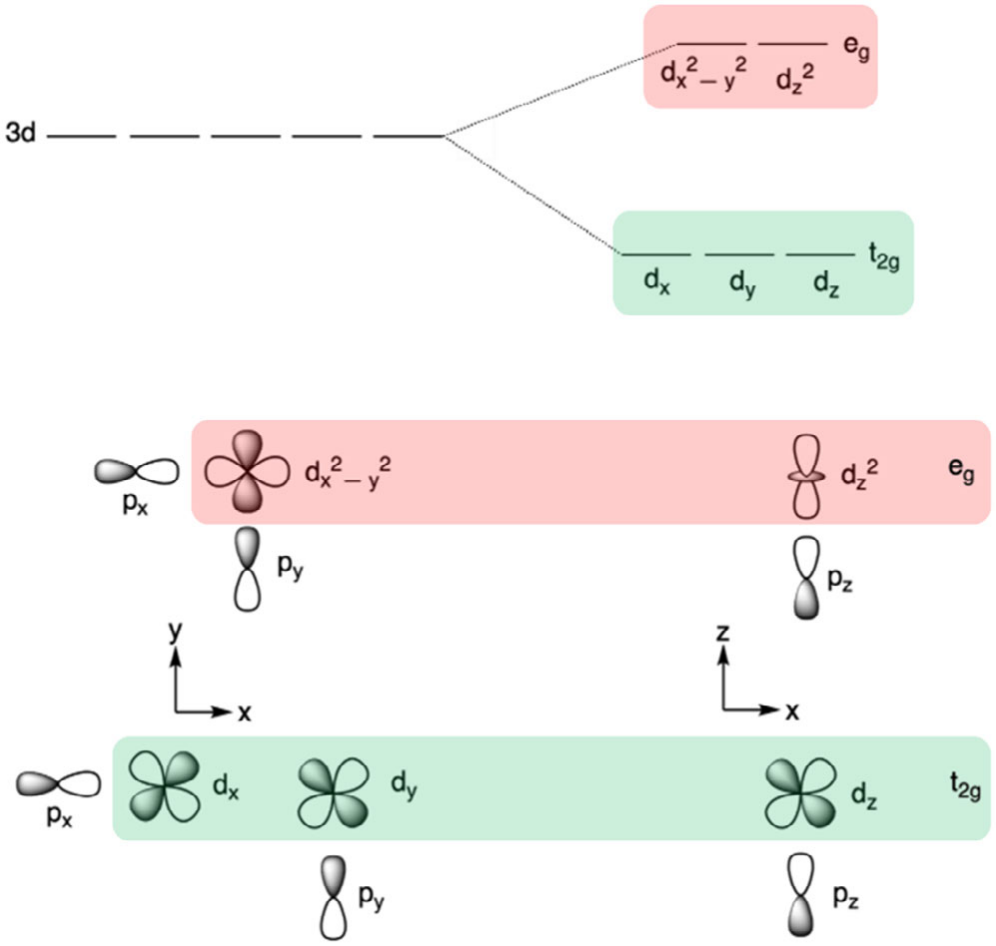
In octahedral fields the 3d orbitals of first‐row transition metals are split into two degenerate sets: t_2g_ (green) consists of the off‐axis *d*_*x*_, *d*_*y*_, and *d* *_z_* orbitals, *e*_g_ (red) consists of the axially aligned *d*_*x*_^2^_‐*y*_^2^ and *d* *_z_* ^2^ orbitals. Oxygen 2p orbitals of OER intermediates have good overlap with the σ‐bonding *e*_g_ orbitals, but poor overlap with the π‐bonding t_2g_ orbitals.

In perovskite oxides, the B‐site metal forms an octahedron through coordination with six oxygen atoms. The d‐orbitals of the TM combine with the 2p orbitals of the adjacent oxygens, forming π‐bonding and π*‐antibonding orbitals (where the O 2p orbitals have weak spatial overlap with the metal *d* *_xy_*, *d* *_xz_*, and *d* *_yz_* orbitals) and σ‐bonding and σ*‐antibonding orbitals (where the O 2p orbitals overlap strongly with the metal $d_{x^{2}-y^{2}}$ and *d*_*z*2_ orbitals) (Figure 8). Degenerate *d* *_xy_*, *d* *_xz_*, and *d* *_yz_* orbitals are collectively called the t_2g_ orbitals, whereas $d_{x^{2}-y^{2}}$ and *d*_*z*2_ are known as *e*_g_ orbitals, with the three t_2g_ orbitals lower in energy than the two *e*_g_ orbitals [125]. Suntivich and Shao‐Horn state that the filling‐status of the TM *e*_g_ orbitals, is an important descriptor for OER catalysis, with an ideal *e*_g_ occupancy of 1.2 [126]. This is due to the good overlap between the σ‐bonding, axially aligned $d_{x^{2}-y^{2}}$ and $d_{z^{2}}$ orbitals of the TM and the oxygen 2p orbitals of the OER intermediates, which promotes electron transfer between the metal cation surface and the adsorbed intermediates, as opposed to the poor overlap between the π‐bonding metal d_*x*_, d_*y*_ and *d* *_z_* orbitals and the O 2p orbitals. The adsorption of the OER intermediates will be stronger with less *e*_g_ filling and weaker with more *e*_g_ filling [45].

**FIGURE 9:**
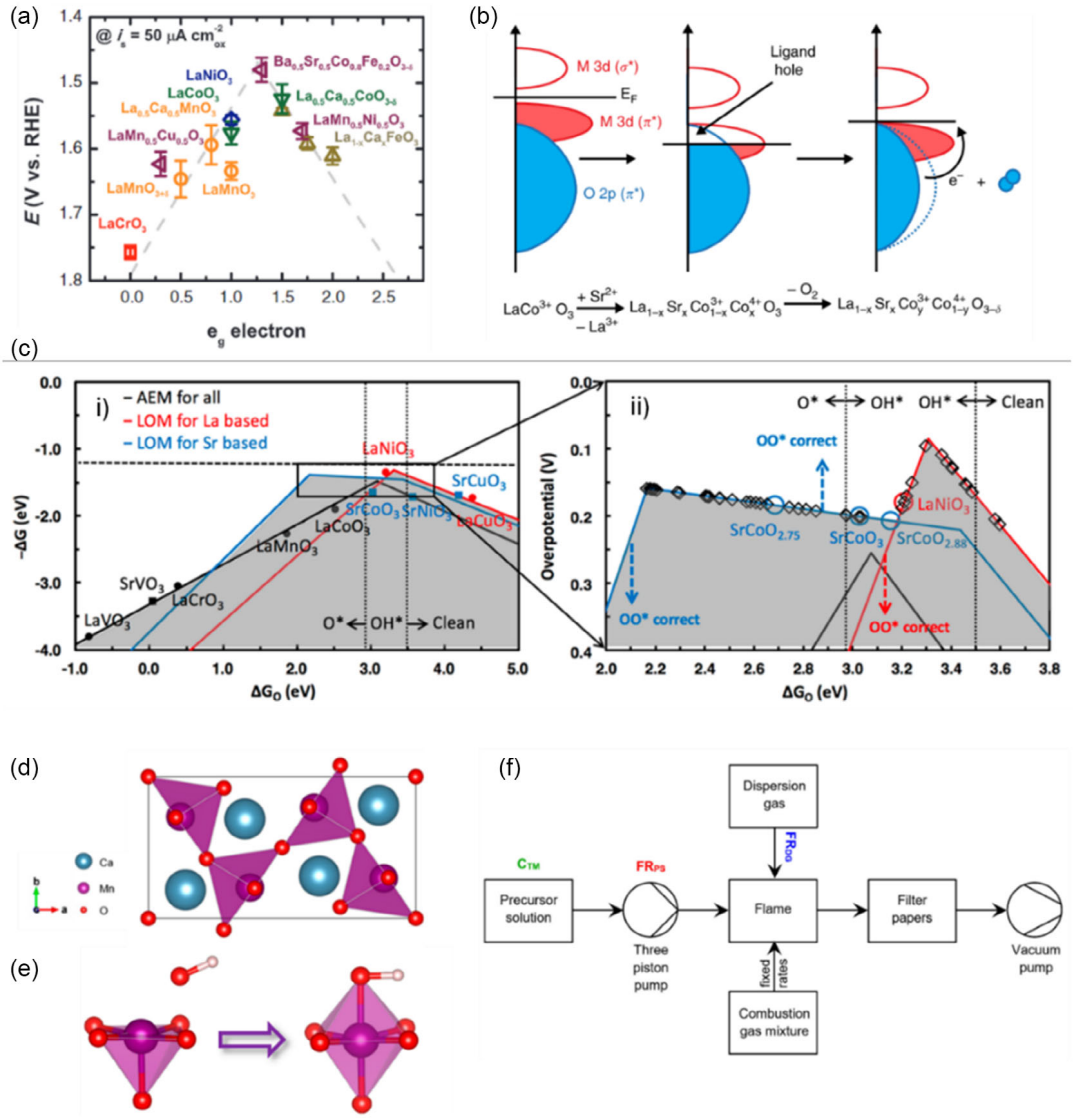
(a) OER activity against occupancy of the *e*_g_ electron of the transition metal, reproduced with permission [126], Copyright 2011, The American Association for the Advancement of Science; (b) relationship between Co—O bond covalency and oxygen vacancy concentration, reproduced from [115] under the Creative Commons CC BY licence; (c) volcano plots of La‐based and Sr‐based perovskites. Black line shows OER activity via AEM for all perovskites, red line shows LOM activity for La‐based perovskites, blue shows LOM activity for Sr‐based perovskites, adapted with permission [43], Copyright 2018, American Chemical Society; (d) unit cells of Ca_2_Mn_2_O_5_ showing ordered oxygen vacancy along directions normal to the ab plane, (e) B‐site cation is more exposed to hydroxide ion interaction in oxygen‐vacant BO_5_ octahedra, adapted with permission [128], Copyright 2014, American Chemical Society; and (f) process diagram of flame spray (FS) synthesis setup, reproduced from [129] under the Creative Commons CC BY licence.

Building on the theoretical work of Hammer and Nørskov [127], in which the centre of the TM d‐band was used as the descriptor for activity on the metal surface, Suntivich and Shao‐Horn investigated the activity of a group of perovskite oxides with the structure A_1–*x*_A′_*x*_B_*y*_B′_1–*y*_O_3_ [126]. It was proposed that the *e*_g_ orbital occupancy of the B‐site TM would have a significant influence on the binding of OER intermediates and thus on OER activity, with the highest activity exhibited when *e*_g_ occupancy is close to unity and oxygen bond character is highly covalent. Defining activity as the overpotential required to induce a current of 50 μA cm^−2^, plotted against *e*_g_ electron occupancy, yielded a volcano plot (Figure 9a) which allowed for the prediction of the high OER activity of Ba_0.5_Sr_0.5_Co_0.8_Fe_0.2_O_3–δ_ (BSCF, where δ denotes oxygen vacancy content and has a value ∼0.5), whose *e*_g_ occupancy is close to one. Indeed, it was found experimentally that the intrinsic activity exhibited by BSCF was at least an order of magnitude higher than that of the state‐of‐the‐art iridium oxide catalyst; a similar relationship has been observed for spinel metal‐oxides [130].

#### 4.2.2 B—O Bond Covalency

Another activity descriptor is the covalency of the M—O bond between the B‐site metal and the lattice oxygen, with more covalent compounds exhibiting increased LOM participation [34, 115]. In general, increasing the oxidation state of the B‐site cation increases the B—O covalency because the metal is more electron‐poor and its empty or partially filled 3d orbitals lie lower in energy relative to the O 2p band. This change in oxidation state can be achieved either through direct substitution of the B‐site cation with a cation of different valence, or through A‐site substitution with a lower valence cation, which can result in oxidation of the B‐site cation to balance the overall charge of the structure [115, 131]. Work by Grimaud et al. found that lattice‐oxygen participation occurs in perovskites that are highly covalent but not in perovskites with a less covalent nature [34]. Furthermore, it was noted that the highly covalent oxides exhibited pH dependent OER behaviour, whereas less covalent oxides displayed non‐pH dependent behaviour; the latter observation would be expected in accordance with the conventional OER mechanism. Mefford et al. also found a direct correlation between covalent bond character and OER activity [115]; in a study of a series of cobaltite perovskites the covalency of the Co—O bond was controlled by the substitution of La^3+^ with the lower valence Sr^2+^ ion in La_1−*x*_ Sr_*x*_CoO_3−δ_ with the authors reporting an increase in oxygen vacancy concentration and improved OER activity with higher Co—O bond covalency. This has been attributed to greater overlap between the d orbitals of the B‐site metal and the s and p orbitals of the O^2‐^ ion as the B‐site oxidation state is increased due to A‐site substitution, which leads to the formation of three different *π* ^*^ and *σ* ^*^ bands [132, 133]. When there is sufficient overlap, the metal 3d *π* ^*^ band and the oxygen 2p *π* ^*^ band hybridise, creating ligand holes, and the Fermi energy can be adjusted by applying an electric potential, allowing for the redox behaviour of lattice oxygen species (Figure 9b). These findings are supported by DFT studies, which have shown that substituting lower valence ions into the A‐site of the perovskite moves the Fermi level closer to the O 2p‐band centre, resulting in improved OER activity; however, moving the O p‐band centre too close to the Fermi level results in decreased stability of the oxide [67]. While increased B—O covalency generally lowers OER overpotential by optimising intermediate binding and enabling LOM participation, it creates a stability trade off. Highly covalent perovskites, with an O 2p band centre close to the Fermi level, facilitate hole‐mediated lattice oxygen redox [134], however, this also lowers oxygen vacancy formation energies and promotes surface amorphisation, cation leaching, and structural degradation at prolonged OER potentials.

### 4.3 Catalyst Design Strategies

The above discussion treats *e*_g_ occupancy and B—O covalency as separate activity descriptors that need to be tuned in order to optimise the adsorption energies of MOH, MO, and MOOH intermediates. However, Hong et al. performed a rigorous statistical analysis of 51 different perovskite oxides reported in literature and showed that there is a strong interrelation between these two values [135]. As such, the O 2p band centre emerges as a particularly useful parameter, because it implicitly governs both *e*_g_ filling and M—O covalency. Consequently, the rational design of catalyst materials often aims to position the O 2p band in an optimal window through approaches including cation substitution, strain engineering, and defect engineering. The flexibility of the perovskite structure allows for the augmentation of the physical, electronic and structural properties of the material through the introduction, or indeed exclusion, of atoms in the crystal structure, allowing the properties to be tuned toward specific activity descriptors. Each of the A‐, B‐, and X‐sites of the perovskite can be modified through doping with various elements (the X‐site contains oxygen in the case of perovskite oxides and will thus be referred to as the O‐site henceforth). Because the A‐site cations tend to be closed‐shell ions with a fixed valence, they are not directly responsible for the chemical and physical properties of perovskites, unlike the BO_6_ octahedra, which is the root of many of the different properties exhibited by perovskites due to the interaction and hybridisation of the orbitals of the B cations and the six surrounding O anions [114]. Instead, A‐site doping is generally used to create a charge imbalance that requires compensation through a change of valence of the B‐site cation, which alters bond covalency [34, 115], and/or by inducing oxygen vacancies, which improves the electronic conductivity of the material and promotes LOM by enabling oxygen mobility [136, 137]. B‐site doping, on the other hand, directly affects the material's electronic structure [80, 126] and can produce synergistic effects between certain metals, such as Ni and Fe [78, 138].

Exploiting the differences in electronegativities, ionic radii and chemical properties of various dopant atoms to strategically select which element to dope into which site can be used to affect the lattice strain, defect content, and morphology of the material and thus impact its chemical, electronic, and physical properties.

Despite their compositional flexibility and promising intrinsic OER activity, perovskite oxides also suffer from several important drawbacks under alkaline OER conditions. Firstly, many compositions exhibit pronounced structural and chemical instability, with potential‐induced surface reconstruction, amorphisation and partial phase segregation occurring already at modest current densities [139, 140, 141]. While such reconstruction can sometimes generate highly active oxyhydroxide‐like surface layers [53, 142], it also complicates the identification of the true active sites and can ultimately lead to mechanical degradation and loss of electrical connectivity [41, 142]. Second, the bulk electrical conductivity of many perovskites remains limited, especially for wide‐band‐gap or strongly distorted structures, necessitating either high loadings of conductive additives or thin, fragile catalyst layers in practical electrodes [103, 104, 143]. Finally, A‐site and B‐site cation dissolution into concentrated KOH is frequently observed, particularly under conditions where lattice oxygen participates in the reaction [42, 53, 142, 144]. Such dissolution not only depletes active sites and alters the surface chemistry but can also contaminate the electrolyte or downstream components. Together, these issues mean that perovskite‐based catalysts often fall short of the stability and activity benchmarks already achieved by simpler TM‐based materials, such as NiFe oxyhydroxides [13, 17], and they motivate the need for careful compositional design approaches.

#### 4.3.1 Strain Engineering

Strain engineering can be achieved through tuning the bond length between the B‐site metal and the lattice oxygen (B—O) and/ or B—O—B bond angle distortion by the substitution of a different‐sized cation into the lattice structure or by an induced lattice mismatch from substrates or defects such as vacancies, holes or dislocations. Such modifications can result in alterations to the electronic structure of the material and can produce significant changes in the adsorption ability of a catalyst's active sites [145]. Tensile strain occurs when the unit cell length is lengthened, while compressive strain occurs when the cell length shortens. Compressive strain leads to a widening of the d‐band, due to the increased overlap between the metal–oxygen orbitals, while tensile strain leads to d‐band narrowing because of the reduced atomic orbital overlap [146]. The B—O—B angle has also been reported to affect the d‐band, with deviations from linearity increasing the electronic resistivity, induced by the reduced metal–oxygen orbital overlap [147]. Moreover, tensile strain can aid the formation of oxygen vacancies due to the reduced oxygen vacancy formation energy from the O 2p band centre being closer to the Fermi level when compared to the unstrained state [148, 149]. Petrie et al. demonstrated that a weakening of M—O chemisorption induced by compressive strain as small as −1.2% in the perovskite LaNiO_3_ resulted in a subsequent increase in both OER and oxygen reduction reaction (ORR) activities to a level above that of the best performing PGM catalyst, with a linear relationship between changes in the d‐band centre and the M‐O chemisorption energy [150]. Bond length can also affect the 3d orbital occupancy, breaking the degeneracy of the *e*_g_ orbitals; compressive strain enhances the electron occupancy of the out‐of‐plane $d_{z^{2}}$ orbital, due to the lowering of the orbital's energy relative to the $d_{x^{2}-y^{2}}$ orbital, while conversely, tensile strain increases $d_{x^{2}-y^{2}}$ orbital occupancy [151, 152, 153]. This is showcased in the work of Fernandez et al. [154], who induced varying degrees of epitaxial strain in La_0.5_Sr_0.5_CoO_3_ and La_0.8_Sr_0.2_Co_0.2_Fe_0.8_O_3_ thin films, grown by pulsed laser deposition (PLD) on different substrates, to probe the effect that different types of strain have on OER reactivity. It was established that biaxial compressive strain suppressed the OER activity of the material while biaxial tensile strain enhanced the oxygen reactivity. Through the use of X‐ray absorption and linear dichroism (XLD) experiments, the energy of the $d_{x^{2}-y^{2}}$orbital was found to shift, relative to the $d_{z^{2}}$ orbital, as the biaxial strain was varied; compressive strain resulted in an increase in the relative energy of the $d_{x^{2}-y^{2}}$ orbital while tensile strain led to a decrease in its relative energy. This breaking of the *e*_g_ degeneracy results in electrons preferentially occupying the lower‐energy orbital, reducing the occupancy of the higher‐energy orbital. OER activity was found to correlate strongly with the occupancy of the $d_{z^{2}}$ orbital, with activity increasing as *d*_*z*_^2^occupancy decreased from near 50% at zero strain to ≈30% when under tensile strain. It is suggested that this could help to further explain the results presented by Petrie et al. [150], as the low‐spin configuration of Ni^3+^ in LaNiO_3_ (t_2g_^6^e_g_^1^) would have $d_{z^{2}}$ orbital occupancy of around 25% at zero strain, which could be increased up to the optimal value of ≈30% with the application of compressive strain. Qi et al. found that the OER activity of La_2/3_Sr_1/3_MnO_3_ (LSMO) as a function of lattice strain exhibited a volcano‐type trend [155]. By depositing LSMO on different single‐crystal substrates to produce different lattice strains, they found that tensile strain lowered the formation energy of oxygen vacancies in LSMO, with bandgap enlargement induced by oxygen vacancies being the dominating factor for OER activity under tensile strain, while the shift of the d‐band centre is more influential under compressive strain. However, the surface geometry of the LSMO thin film was later found to play a more essential role in OER activity than strain [156].

#### 4.3.2 Vacancy Concentration

Oxygen vacancies can both improve the ionic conductivity of materials and reduce the adsorption energy barriers of the reaction intermediates through the tuning of the electronic structure [157, 158]. Defects at the O‐site cause the TM d‐band centre to shift relative to the O 2p band, resulting in an increase in M 3d/O 2p orbital hybridisation and bond covalency [159] and subsequently better catalytic performance. However, too many oxygen vacancies may also cause a shift in the O 2p band centre relative to the Fermi level, which can have a detrimental effect on catalytic performance [160]. The concentration of oxygen vacancies in perovskite structures can be systematically tuned through compositional engineering, particularly through A‐site substitution of lower‐valence cations; for example, in the La_1‐*x*_Sr_*x*_CoO_3‐δ_ system, substitution of La^3+^ with Sr^2+^ requires charge compensation through either oxidation of Co cations or creation of oxygen vacancies [115]. On the other hand, in perovskites with an A‐site cation deficiency, charge balance has been shown to be achieved primarily through the formation of oxygen vacancies rather than the oxidation of low‐valence B‐site cations, resulting in enhanced ionic conductivity [131, 161]. The LOM is reliant on the reversible formation and refilling of oxygen vacancies, and inducing oxygen deficiencies in spinel and perovskite oxides has been shown to increase the LOM behaviour of these materials [54, 55, 57, 162]. This is because the critical intermediate distinguishing LOM from AEM is the OO* species bound to the metal site via the original adsorbed oxygen atom, with a surface oxygen vacancy present. Using ab initio computations, Rong et al. showed that as the bulk stability of ABO_3_ decreases, the OER mechanism transitions from AEM to LOM, due to the increased favourability of oxygen vacant intermediates as the electron donation by the B‐site cation becomes more ineffective [54, 163]. Liu et al. have demonstrated, by synthesising the series of perovskite oxides Sr_1‐*x*_Ca_*x*_Co_0.5_Fe_0.5_O_3‐δ_ (where *x* = 0, 0.5, and 1, denoted as SrCF, CaSrCF, and CaCF, respectively), that increasing oxygen vacancy concentration promotes the LOM through increased lattice oxygen migration, higher vacancy ordering was found to reduce oxygen diffusivity and imped LOM, verified by oxygen intercalation measurements [164]. In the cubic structure of SrCF, the oxygen vacancies were randomly distributed throughout the structure, whereas in the ordered orthorhombic structure of CaSrCF, composed of alternating tetrahedral (Co/FeO_4_) and octahedral (Co/FeO_6_) layers, the low oxygen concentration within the tetrahedral layers led to the formation of oxygen vacancies within these layers. In contrast, the tetrahedral layers in CaCF had alternating orientation, leading to the oxygen vacancies being distributed across every other layer with a high degree of ordering, as has been seen in other studies [165, 166]. Through the use of XPS, it was found that, although the A‐site cations remained in the +2 oxidation state, the introduction of Ca^2+^ into SrCF reduced the oxidation states of the B‐site Co and Fe cations, which induced oxygen vacancies to maintain charge neutrality. As XPS cannot give reliable information on oxygen vacancies, the oxygen non‐stoichiometric value (δ) was determined through iodometric titration and corroborated the trend conjectured from the XPS data [164]. These findings are similar to those previously reported by Yoo et al., who computationally investigated the effect of subsurface oxygen vacancies on lattice oxygen participation for LaMO_3‐δ_ (M = Ni, Co, Cu) [136]. Introducing oxygen vacancies into the subsurface layer increased both reaction energies and activation barriers for surface oxygen participation, as extracting additional surface oxygen becomes more difficult from oxygen‐deficient structures. However, this effect was much smaller for late TMs (LaCuO_3‐δ_ < LaNiO_3‐δ_ < LaCoO_3‐δ_), demonstrating that metals with high electronegativities are better able to stabilise the system in the electron‐rich environment created by vacancies, relative to metals with lower electronegativities. This finding suggests an optimal vacancy concentration exists: sufficient to enable facile oxygen mobility, but not so excessive as to destabilise further oxygen extraction. A further study by the same authors used the binding energy of atomic oxygen (Δ*G*_O_) to predict the OER mechanisms and activity of perovskites [43], yielding a volcano‐type plot (Figure 9c) with three categories emerging: (1) strongly binding perovskites such as LaCrO_3_ (Δ*G*_O_ = 0.38 eV) and SrVO_3_ (Δ*G*_O_ = 0.05 eV), which prefer AEM (left side of the volcano plot); (2) moderately binding perovskites (2.0 < Δ*G*_O_ < 3.0 eV), such as LaNiO_3_ (Δ*G*_O_ = 3.21 eV) and SrCoO_3_ (Δ*G*_O_ = 3.03 eV), where AEM and LOM compete (around the peak of the volcano plot); and (3) weakly binding perovskites (Δ*G*_O_ < 2.0 eV), such as LaCuO_3_ (Δ*G*_O_ = 4.38 eV) and SrCuO_3_ (Δ*G*_O_ = 4.18 eV), which strongly favour LOM (right side of the volcano plot). Interestingly, the top of the LOM volcano for Sr‐based perovskites is considerably broader than that of the LOM volcano for La‐based perovskites, suggesting that more moderately binding Sr‐based materials can proceed OER via the LOM route. Oxygen‐deficient perovskites, with the general stoichiometry A_2_B_2_O_5_, contain ordered defects in their crystal structures and offer improved activity when compared to their oxygen‐abundant counterparts. Kim et al. examined the electrocatalytic performance of the oxygen‐deficient perovskite oxide Ca_2_Mn_2_O_5_, prepared from the standard perovskite oxide CaMnO_3_ through a reductive annealing process [128]. This resulted in the B‐site octahedra adopting a square‐pyramidal geometry, with an oxygen atom missing along directions normal to the *ab* plane, giving a zigzag structure that resulted in molecular‐level porosity (Figure 9d). The claim of molecular level porosity was inferred from Rietveld refinement of XRD data, although a lack of gas adsorption/ desorption isotherms means that a direct link between this crystallographic data and the mesoporosity of the material could not be drawn. Compared to the perovskite CaMnO_3_, Ca_2_Mn_2_O_5_ exhibited higher OER activities, with an OER mass activity of 30.1 A/g at 1.70 V (vs. RHE), almost 6 times higher than that of CaMnO_3_, which had an OER mass activity of 5.19 A/g at the same potential. The B‐site cation is considered to be an active site for OER, meaning that oxygen vacancies resulting in a BO_5_ octahedra can leave the B‐site cation more accessible to the absorbate, thus enhancing the adsorption capability of the catalyst (Figure 9e) [128].

#### 4.3.3 Surface Layer Reconstruction

The active sites of metal oxide catalysts are traditionally considered to be the metal centres, although the local and electronic structure of perovskite materials has been shown to change during oxygen evolution conditions [139, 140]. This is likely due to the simultaneous occurrence of both AEM and LOM, which can often lead to the formation of a surface layer that is different to that of the bulk oxide; Fabbri et al. first reported the dynamic reconstruction of this surface layer as being key for highly active catalysts [53]. It has been observed that the surface of BSCF and other perovskite oxides with similarly high OER activity quickly become amorphous under OER conditions, while less active perovskites do not undergo this rapid amorphisation [141]. This observation has been rationalised using molecular orbital theory, with the hypothesis that it was due to a Fermi level pinning effect caused by the closer proximity of the O p‐band centre to the Fermi level in perovskites such as BSCF when compared to the less active perovskites. An O p‐band level higher than −2.2 eV relative to the Fermi level has been proposed as being necessary to induce amorphisation (having high O p‐band centres has been directly correlated to a high concentration of oxygen vacancies and high lattice‐oxygen mobility in perovskite‐oxides such as BSCF) [167]. Fabbri et al. have directly attributed the formation of this amorphous layer to the LOM [42, 53, 142] and used a flame spray (FS) method (Figure 9f) to synthesise a BSCF nano‐catalyst with Co cations in a reduced oxidation state, resulting in a higher concentration of oxygen vacancies (δ∼0.75) and improved formation of the highly OER active amorphous (oxy)hydroxide layer [53]. The catalyst was monitored via operando X‐ray absorbance spectroscopy (XAS) measurements, and the layer was found to form due to the irreversible oxidation of Co at anodic potentials (a finding supported by Cao et al.) [168], while Fe was not observed to have undergone any oxidation state change [142]. La_0.2_Sr_0.8_CoO_3‐δ_ (LSC) was also found to undergo this operando oxidation state change of Co, although the initial oxidation state of Co in LSC was near +3 compared to around + 2 for BSCF; meanwhile, CoO (also prepared via FS and with a similar Co oxidation state to BSCF) only showed a slight increase in oxidation state. This suggests that the electronic structure of perovskite OER catalysts can change significantly under OER conditions and indicates that the LOM is the fundamental process behind the formation of the (oxy)hydroxide surface layer, an assertion supported by ICP‐OES measurements carried out on the electrolyte solution post‐testing, which showed significant A‐site cation dissolution but minimal B‐site cation leaching, thus leaving behind a Co/Fe‐enriched surface layer [53]. Interestingly, recent further refinement of the FS synthesis method has shown that the flow rate of the precursor solution (FR_PS_), flow rate of the dispersion gas (FR_DG_), and total metal concentration (*C*_TM_) all influence the OER activity of BSCF, as well as the Co and Fe oxidation state, suggesting that these parameters can also be used to control the oxygen vacancy content in BSCF [129].

#### 4.3.4 Morphology Control

The number of available active sites can be increased by engineering the surface morphology of the catalyst to have a high surface area. Various perovskite materials with novel morphologies have been reported as being excellent OER catalysts, including coral [169], yolk‐shell [170], and urchin‐like [171]. Reducing the catalyst particle size leads to an increase in the surface area to volume ratio, making the use of nanomaterials particularly appealing; these can include nanospheres [172], nanorods [173], nanofibers [174], and nanotubes [175]. The choice of synthesis approach can have a significant impact on the resulting morphology of the material, with the hydrothermal and sol–gel methods being the most commonly reported methods for producing nanoscale materials with high surface area (Section 5).

## 5 Perovskite Synthesis Techniques

**FIGURE 10:**
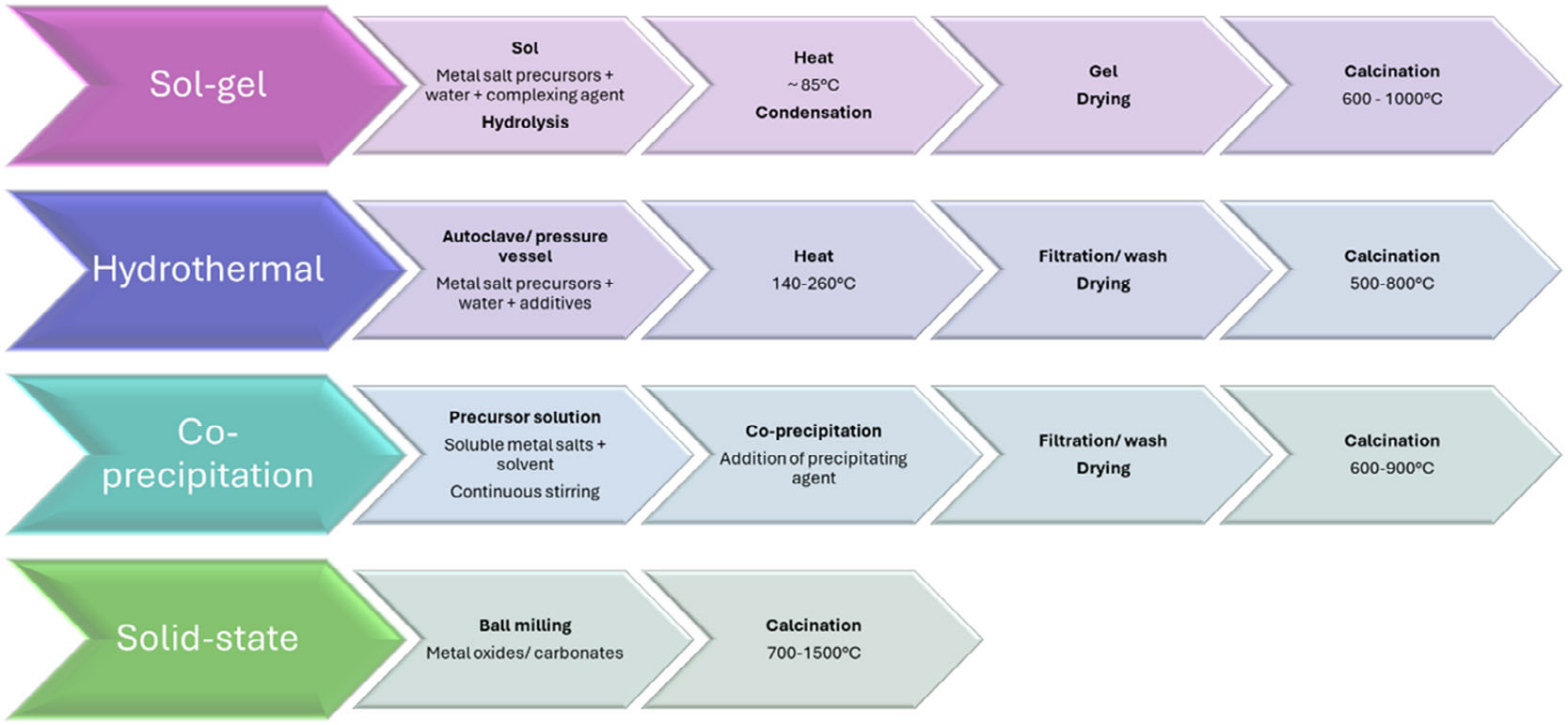
The synthesis processes for the four main routes to preparing perovskite oxide materials.

Material preparation procedures can be divided into solid‐state, liquid‐phase, and gas‐phase synthesis. Solid state is generally used for the synthesis of bulk materials, liquid‐phase allows for the production of nanomaterials and gas‐phase is generally utilised for thin film production [176]. Solid‐state and liquid‐phase synthesis commonly produce powders which can then be used to coat electrodes for electrolysis. Gas‐phase, including FS, physical vapour deposition (PVD), and magnetron sputtering techniques are mainly used for electronic devices and photocatalyst preparation, so will not be covered in detail here. The four main types of solid and liquid synthesis approaches used for perovskite water splitting catalyst preparation are sol–gel, hydrothermal, co‐precipitation (all liquid‐state syntheses), and solid‐state synthesis (Figure 10). Crystallographic analysis techniques such as X‐ray diffraction (XRD) are commonly used to check that the desired phases are obtained with good crystallinity and purity after synthesis.

### 5.1 Sol–Gel Method

**FIGURE 11:**
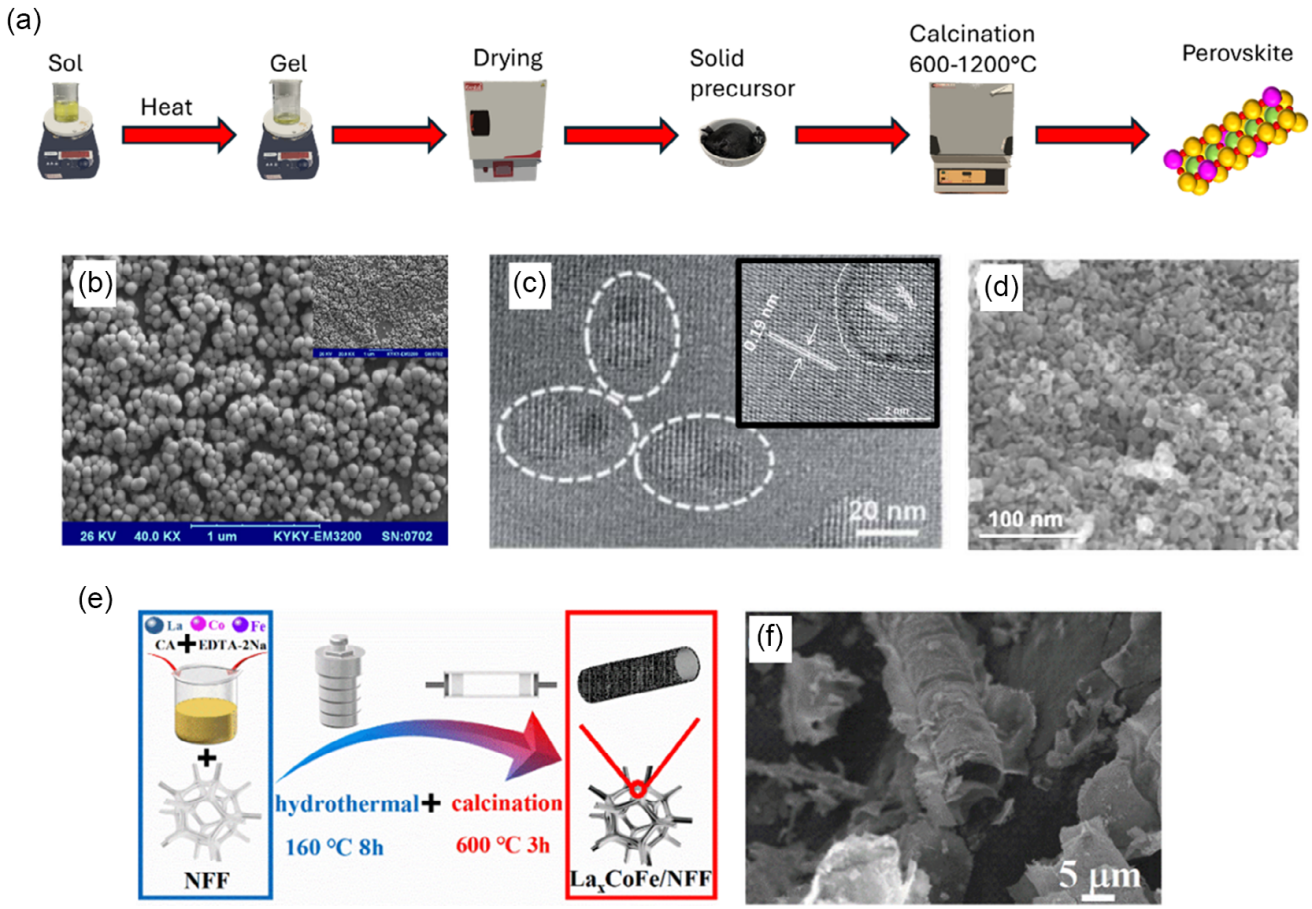
(a) Schematic representation of the sol–gel synthesis method; (b) spherical LaCoO_3_ nanoparticles produced via sol–gel synthesis, reproduced from [172] under the Creative Commons CC‐BY licence; (c) HRTEM image showing nano‐sized pitting in the lattice of the perovskite H‐LFCMO, produced by sol–gel synthesis, reproduced with permission [177], Copyright 2021, Royal Society of Chemistry; and (d) high surface area perovskite LSM prepared via co‐precipitation method, reproduced from [178] under the Creative Commons CC BY‐NC 3.0 licence. (e) schematic diagram of synthesis process for La_*x*_CoFe/NFF and (f) hollow tube structure exhibited by La_*x*_CoFe/NFF, reproduced from [179] under the Creative Commons CC‐BY licence.

The sol–gel method is a facile multi‐step process used to synthesise high‐quality micro and nanostructures, which provides reasonable control over the composition, size and surface properties of materials. The process is typified by the use of a colloidal solution (sol) that transitions into another colloidal system (gel) as a result of chemical reactions (Figure 11a) and can be described in five steps: hydrolysis, condensation, ageing, drying, and crystallisation [75]. There are several variants of the sol–gel method that can be used for the synthesis of perovskites, with the chelate polyesterification approach of the Pechini method being the most popular, due to its simplicity and versatility. In this method, metal nitrate salts are dissolved and mixed with complexing agents such as citric acid, EDTA, etc., which act to bind the metal ions together in a precursor solution [80]. An optimal ratio of complexing agents to metal ions is essential in order to prevent partial metal segregation and precipitates forming in the solution. The metal nitrate/ chelating agent solution is stirred and then heated to form a viscous material, which is subsequently dried to form a solid precursor. This solid precursor can then be calcined to form the perovskite structure; temperatures above 600°C are generally used, with temperatures approaching 1000°C usually resulting in the purest perovskite crystal structure [180, 181, 182, 183], although higher temperatures can lead to sintering, which can affect the porosity and morphology of the final product. Sadabadi et al. used a sol–gel approach to synthesise LaCoO_3_ nanoparticles using glycine as a fuel in a two‐step auto‐combustion approach, which yielded highly crystalline spherical nanoparticles with a size below 100 nm after calcining at 600°C for 2 h (Figure 11b) [172]. Furthermore, Selvadurai et al. synthesised the oxygen deficient perovskite LaFe_0.75_Cr_0.15_Mo_0.10_O_3‐δ_ via the sol–gel method with calcination at 900°C for 2 h and observed, through high resolution (HR) transmission electron microscopy (TEM), porous pits several nanometres across that were highly disordered in comparison to the nearby lattice (Figure 11c); it was hypothesised that this discontinuity of the surface lattice allowed for the creation of oxygen vacancies [177]. When cast onto nickel foam, the material displayed reasonable OER activity, with a η_10_ of 263 mV, although it should be noted that the surface area of nickel foam is significantly higher than that of a planar substrate such as a glassy carbon electrode, meaning that there could be some variation in results if samples were tested on both of these different substrates.

### 5.2 Hydrothermal Method

In hydrothermal synthesis, materials are crystallised directly from aqueous solution, where water acts as both the solvent and the pressure‐transmitting medium [184]. Pressure, temperature and composition are carefully controlled and small changes in these variables can result in significant changes to the crystal structure, size, and morphology of the synthesised material [185]. Reactants are placed in a PTFE‐lined cylinder, which is then sealed in a pressure vessel and placed in an oven, resulting in an increase in temperature and pressure. The pressure is controlled by adjusting both the temperature and the percentage‐filling of the vessel and this method generally allows for the production of crystal phases at temperatures considerably lower than would be possible for a solid‐state reaction; however, production yield is limited by the size of the pressure vessel. The hydrothermal method allows catalysts to be prepared either as powders or grown in situ on substrates such as meshes or foams, and nano‐scale morphology can often be realised [186]. Özbay et al. used the hydrothermal approach to synthesise LaMnO_3_ and LaNiO_3_ perovskite oxides with nanosized spherical particles, noting that a highly phase‐pure powder was difficult to obtain with hydrothermal reaction conditions, but that the usage of a chelating agent during the synthesis could mitigate the formation of impurities in the final product [187]. Recently, Xu et al. used EDTA and citric acid as chelating agents in a hydrothermal synthesis followed by a calcination step that produced an A‐site deficient perovskite La_*x*_Co_0.4_Fe_0.6_O_3‐δ_ with a hollow tube structure grown on nickel‐iron foam (Figure 11e,f) [179]. This approach led to a high‐performance bifunctional OER/HER catalyst that exhibited good performance at both 10 and 100 mA cm^−2^ (234.7 and 296.1 mV, respectively) with 20 h stability at 10 mA cm^−2^. The solvothermal method is closely related to the hydrothermal method; the aqueous solution is replaced with a solvent of a non‐aqueous nature, allowing for the formation of micro‐ and nano‐scale powders and the isolation of structures and compositions that are otherwise inaccessible to conventional high temperature synthesis [188].

### 5.3 Co‐Precipitation Method

The co‐precipitation method refers to a solution containing two or more cations which are uniformly precipitated out of solution in the form of hydroxides, carbonates, oxalates, or citrates. The precipitates are then washed before being calcined at a certain temperature, often between 600°C–900°C [189, 190]. These high temperatures can produce near homogeneous pure phase polycrystalline powders of the desired material. To achieve a high yield of precipitation, the solubility of the compounds used should be similar to each other, while precise control of the concentration, temperature, pH and mixing speed of the precursor solution should be ensured, as these can all affect the morphology and purity of the final product [176]. Nguyen et al. used the co‐precipitation method to synthesise a perovskite oxide with high entropy B‐site consisting of near equimolar ratios of Cr, Mn, Fe, Co, and Ni. The approach led to nanoparticles consisting of a single‐phased orthorhombic perovskite structure, with the optimised catalyst displaying good OER activity, with a η_10_ of 325 mV and a Tafel slope of 51.2 mV dec^−1^ [191]. Huang et al. reported the synthesis of the perovskite La_0.6_Sr_0.4_MnO_3_ (LSM) with a high surface area of 48 m^2^g^−1^ (Figure 11d) via the co‐precipitation method using N, N‐dimethylacetamide (DMAC) as the solvent, chosen due to the similar diffusion rate of metal ions as opposed to an aqueous solution, where metal ions can precipitate out at different rates [178]. Tetramethylammonium hydroxide was selected as the precipitant due to its strong alkalinity, enabling the metal cations to precipitate as hydroxide in the DMAC without introducing additional metal cations such as Na^+^, which are often used in the form of Na_2_CO_3_ [192].

### 5.4 Solid‐State Method

The solid‐state method is one of the most commonly used routes for bulk synthesis of perovskites [193]. This high temperature method generally results in highly crystalline materials with a low yield, low surface area, and large, unevenly distributed particle sizes [176]. Also known as the ‘shake and bake’ method [184], solid‐state synthesis requires oxide and/or carbonate raw materials, which are milled together and then reacted at very high temperature, often in excess of 1000°C [176], to form the desired structure; however, on an atomic scale the reactants are not homogenously mixed, resulting in an intrinsically slow reaction. While not a sophisticated method, solid‐state synthesis is relatively effective for producing large quantities of material from inexpensive precursors.

Table 1 provides a summary of the most commonly used synthesis methods for perovskite electrocatalysts along with their advantages and disadvantages.

**TABLE 1:** Summary of the most commonly used synthesis methods for perovskite OER electrocatalysts.

Synthesis method	Precursors	Calcination temperature	Advantages	Disadvantages
Sol–gel	Metal salts (e.g. nitrates, sulphates, chlorides) Complexing agents (e.g. EDTA, citric acid)	600°C–1000°C	Produces good uniformity of micro‐ and nanoparticles	Sintering can reduce surface area of product Time consuming process
Hydrothermal	Metal salts (e.g. sulphates, chlorides, nitrates) Additives (e.g. ethylene glycol)	500°C–800°C	Low temperature Can produce a range of nano‐scale morphologies	Low yield Defect rich microstructure
Co‐precipitation	Metal salts (e.g. chlorides, nitrates, acetates) Precipitating agents (e.g. ammonia, urea)	600°C–900°C	Low cost Easily scalable	Purity and morphology of product are difficult to control Multiple washes of precipitates are time consuming
Solid‐state	Metal oxides/ carbonates	700°C–1500°C	Low cost Easily scalable	High temperature Low yield Atomic level inhomogeneity Uneven particle size distribution

### 5.5 Other Synthesis Methods

While the above‐mentioned synthesis approaches constitute the majority of perovskite preparation methods regularly reported in literature, there is a growing use of more advanced synthesis methods, which allow finer control over parameters such as morphology, particle size, defect density, microporosity, and cation distribution. For example, the microemulsion method uses a thermodynamically stable mixture of oil, water, and surfactant that confines nucleation and growth to within nanoscopic droplets [194, 195]. The droplet size is influenced by the water‐to‐surfactant ratio and directly affects particle size of the final product [196]; fine tuning of the droplet size enables narrow particle‐size distributions and high surface areas, however, this approach involves the use of complex surfactant systems and multiple washing steps that may be hard to translate into industrial‐scale production. Template‐assisted synthesis is another approach that can be used to achieve precise, nanoscale perovskite structures through the use of a pre‐formed scaffold to direct morphology and can be categorised into either hard or soft templating [195]. In hard templating, a rigid support such as mesoporous silica is impregnated with perovskite precursors and the template is chemically removed after calcination, yielding highly ordered pores or nanotubes with excellent control over size and connectivity. In soft templating, surfactants or polymers such as polyvinylpyrrolidone (PVP) self‐assemble into micelles or nanofibers that guide perovskite nucleation. Here, the templates burn out during calcination, offering simpler, more scalable processing but typically less sharply defined porosity than hard templating; electrospinning is an example of a soft templating technique that is finding increased use in perovskite electrocatalyst synthesis [197]. Microwave synthesis uses dielectric heating to rapidly bring precursor mixtures to reaction temperature, drastically shortening synthesis and calcination times compared to conventional furnaces [195]. In perovskite oxides, this can be implemented as microwave‐assisted sol–gel, coprecipitation, hydrothermal, and microemulsion routes, amongst others, and more recently as ‘microwave shock’ treatments that crystallize or restructure powders within seconds [195, 198]. The fast, volumetric heating can promote finer particle sizes, higher defect concentrations, and metastable phases that are difficult to access thermally; however, scale‐up and uniformity remain significant challenges as non‐uniform microwave absorption can lead to hot spots, inhomogeneous phases, and uncontrolled sintering.

## 6 Recent Developments

### 6.1 La‐Based Perovskites

La‐based perovskites are some of the most promising candidates for large‐scale OER applications, owing to the relative earth abundance of La and the high oxygen mobility of its oxides [159, 199, 200]. There are ongoing discussions about the OER reaction mechanisms for these types of perovskites. LOM participation has been observed in many La‐based perovskites; Yoo et al. have shown that it is, in fact, more feasible than the conventional AEM in LaNiO_3_ [136]. Strongly binding perovskites, such as LaCoO_3_, favour AEM to LOM, while weakly binding perovskites, such as LaCuO_3_, prefer LOM to AEM; as LaNiO_3_ is moderately binding, it can participate in both mechanisms_._ [54, 136, 201] Hou et al. investigated A‐site doping in LaCoO_3_ with different A‐site elements, using a molten salt synthesis to prepare the perovskite series (La_1‐*x*_M_*x*_CoO_3_ (M = Ce, Sr, Ca, Ba, *x* = 0.05, 0.1, 0.2, 0.3, 0.4, 0.6, 0.8) [202]. It was found that the perovskite La_0.9_Ce_0.1_CoO_3_ exhibited the lowest η_10_ value of 343 mV, with XPS analysis suggesting that this is because of the high Co^3+^/Co^2+^ ratio brought about by a charge compensation effect after Ce^4+^ replaces La^3+^ at the A‐site. Simultaneously, the introduction of CeO_2_ as a minor secondary phase after Ce doping also improves the activity, due to the well‐known dynamic redox behaviour of Ce species, resulting in excellent oxygen storage and release capacity; similar observations have been made by Nouri et al. in LaMnO_3_ perovskites [203].

As alluded to earlier, the OER activity of Ni and Co‐based catalysts can be enhanced by the incidental or intentional incorporation of a small amount of Fe, but the root cause of this effect is still under debate [78]. The flexibility and adaptability of the perovskite structure make materials such as LaNiO_3_, LaCoO_3_, and LaFeO_3_ an ideal platform for exploring and further exploiting this synergy.

### 6.2 Fe Incorporation

**FIGURE 12:**
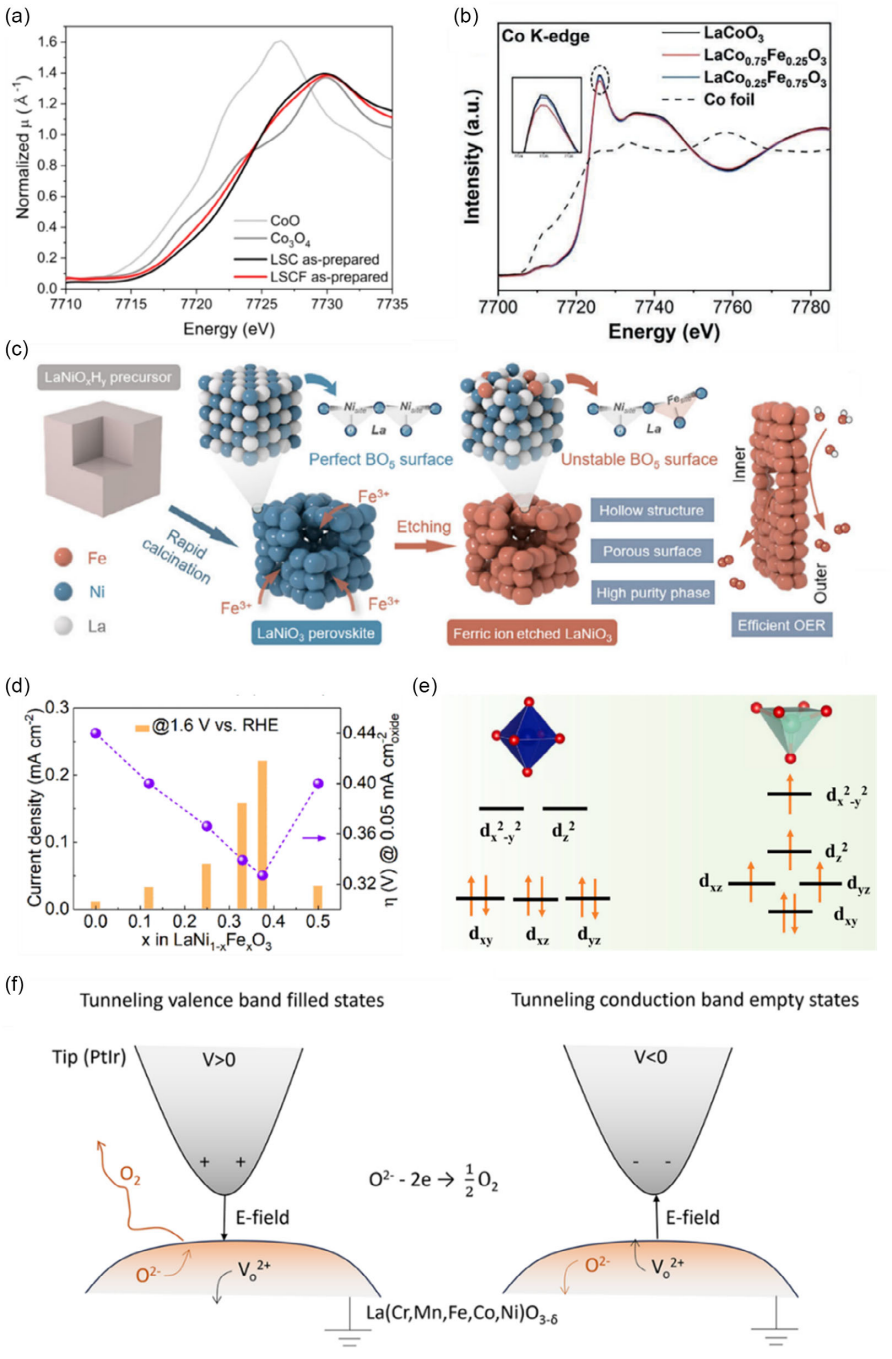
(a) Co K‐edge spectra of LSC and LSCF, reproduced with permission [81], Copyright 2019, American Chemical Society; (b) Co K‐edge XANES spectra of LaCoO_3_, LaCo_0.75_ Fe_0.25_ O_3_, and LaCo_0.25_ Fe_0.75_ O_3_ reproduced with permission [170], Copyright 2022, Wiley; (c) schematic illustration of the synthesis of LNFe^III^‐spe, reproduced with permission [204], Copyright 2025, John Wiley and Sons; (d) comparison of the specific OER activities measured at 1.6 V versus RHE (bars). Overpotentials (η) for LNFO films as required to obtain 0.05 mA cm^−2^ oxide in CV measurements (circles), reproduced with permission [205], Copyright 2021, American Chemical Society; (e) electronic configurations of octahedrally‐coordinated and pyramidally‐coordinated Co ions in BiCoO_3_, adapted with permission [87], Copyright 2023, Elsevier; and (f) illustration of the tip bias leading to oxygen extraction from the lattice and mobility of oxygen vacancies in the HEPO surface, a positive bias voltage enables oxygen extraction, reproduced from [206] under the Creative Commons CC‐BY 4.0 licence.

It has been widely reported that incorporating even a small amount of the element Fe into TM‐based materials, particularly those that contain Co and/or Ni, can significantly improve the OER activity of these materials [16, 78, 138], and this approach has been widely investigated in TM‐based perovskites. For instance, Kim et al. investigated the functional role of Fe in enhancing the OER activity of the LaCoO_3_‐derived perovskite LSC [81]. Fe was incorporated into the B‐site of LSC in a Co:Fe ratio of 8:2, giving La_0.2_Sr_0.8_Co_0.8_Fe_0.2_O_3‐δ_ (LSCF), which was found to exhibit ~6 times higher current density than LSC with improved stability. X‐ray absorption near edge structure (XANES) measurements (Figure 12a) found that the valence state of Co in both LSC and LSCF was close to + 3, however the Co K‐edge of LSCF was positioned ~0.3 eV lower than in LSC, suggesting a slightly lower valence state for Co in LSCF. In operando XANES measurements showed that during anodic polarisation the Co K‐edge of both LSC and LSCF were similar, meaning that they were oxidised to the same valence state. However, because the initial Co K‐edge position of LSCF was lower than LSC, it exhibited a greater average increase in Co oxidation state. Meanwhile, it was found that the Fe oxidation state in LSCF did not undergo any significant change, suggesting that the Fe does not act as an active site for the OER. In contrast to this, XANES measurements by Bao et al. (Figure 12b) found no obvious shift of the Co K‐edge of LaCoO_3_‐derived LaCo_0.75_Fe_0.25_O_3_ or LaCo_0.25_Fe_0.75_O_3_ [170], although it was found that the Fe K‐edge of LaCo_0.75_Fe_0.25_O_3_ was shifted significantly to lower energy compared to LaCo_0.25_Fe_0.75_O_3_, indicating a decrease in the Fe valence state, rationalised by an increase in oxygen vacancies. However, in situ Raman spectroscopy suggested that Fe substitution induces surface reconstruction at lower applied potentials through the pre‐oxidation of Co sites. Zhao et al. employed a novel ferric etching strategy to the perovskite LaNiO_3_ that corroded weaker La—O bonds at the terminal sites of the crystal structure to form La/O vacancies, while the ferric ions also underwent displacement reactions with surface nickel, creating additional active sites (Figure 12c) [204]. Starting with LaNiO_*x*_H_*y*_ nanocubes synthesised via the hydrothermal method, a high temperature calcination step was used to produce porous hollow nanocubes of LaNiO_3_; the etching process was then carried out by immersing these LaNiO_3_ nanocubes in a solution of ferric nitrate. The resulting material was named LNFe^III^‐*spe*. In situ XAS and Raman spectroscopy showed that the LNFe^III^‐*spe* underwent surface reconstruction at a significantly lower potential than the unetched LaNiO_3_, likely primarily due to La vacancies bringing the O 2p centre closer to the Fermi level and facilitating LOM. Ni/Fe ratios from XPS data indicated that the ferric ions primarily replace Ni atoms, the XRD pattern of LaNiO_3_ was unchanged before and after etching, suggesting that this change may only be affecting the surface at a sub‐nanometre scale. Electrochemical testing showed an η_10_ of 280 mV for LNFe^III^‐*spe*, 140 mV lower than the unetched LaNiO_3_, with a decay rate of 0.22 mV h^−1^when tested over 300 h at 10 mA cm^−2^.

Cheraparmbil et al. have used in situ electrochemical surface‐enhanced Raman spectroscopy and electron energy loss spectroscopy to investigate the active centres in a series of perovskites derived from LaNiO_3_ and LaFeO_3_ [207]. While the activity of LaNiO_3_ and LaFeO_3_ was found to arise from surface oxyhydroxides formed in situ from the active centres, NiOOH and FeOOH, respectively, it was found that in the mixed metal LaNi_0.5_Fe_0.5_O_3_, the active centre was exclusively Fe, forming a combination of FeOOH and Fe‐O‐Ni species. It was noted that the activity of LaFeO_3_ was increased by the interaction of the active centre with Ni impurities in the electrolyte, oxidising to form Fe^4+^=O species, however Fe leaching hindered stability over longer periods; it was postulated that LaNi_0.5_Fe_0.5_O_3_ offered a good balance between Fe dissolution and the formation of stable active sites, leading to improved OER performance compared to LaNiO_3_ and LaFeO_3_. The LaNiO_3_/LaFeO_3_‐derived series LaFe_*x*_Ni_1‐*x*_O_3_ (LNFO) has been studied in some depth due to the high OER activity and relative low cost of the materials. L. Wang et al. studied epitaxial thin films of LNFO and found that Fe substitution alters the valence state of Ni, resulting in a volcano‐like OER activity trend with *x* = 0.375 at the vertex (Figure 12d) [205]. Through the use of in situ XPS and ex situ XAS, it was found that Fe substitution induces electron transfer from Fe to the Ni sites, oxidising Fe from Fe^3+^ to Fe^4+^ and reducing Ni from Ni^3+^ to Ni^2+^. The increased oxidation state of the Fe resulted in an increase in the width of the TM 3d band and enhanced the TM 3d–O 2p hybridisation, resulting in increased OER activity, while both Fe^3+^/Fe^4+^ and Ni^2+^/Ni^3+^ ratios were found to increase as Fe content increased, corroborated by Idrees et al. [208], leading to larger lattice parameters due to the longer B—O bond lengths for Ni^2+^ and Fe^3+^.

H. Wang et al. synthesised nanorods of LNFO using a hydrothermal preparation method, reporting the optimal Ni:Fe ratio as 8:2 [173]. The optimised LNFO material achieved an overpotential of 302 mV at 10 mA cm^−2^, with a Tafel slope of 50 mV dec^−1^, and only displayed a slight decrease in current after 20 h of testing. Of the three different catalysts produced (*x* = 0.1, 0.2, 0.5), the 8:2 Ni:Fe ratio was found through Brunauer–Emmett–Teller (BET) analysis to have the highest specific surface area at 58.1 m^2^ g^−1^; this was able to partially account for the improved OER activity, while XPS spectra showed an upward shift of the metal *d*‐band towards the Fermi level (*E*_F_) compared to the undoped LaNiO_3_, which promotes electron transfer and thus boosts activity. Q. Wang et al. also reported an optimal Ni:Fe ratio of 8:2 in Fe doped LaNiO_3_ [169]; using a sol–gel method, they synthesised LNFO (*x* = 1.0, 0.7, 0.5, 0.3, 0.2, 0) with a calcination temperature of 700°C for 6 h and confirmed the perovskite structure through XRD analysis, with increasing Fe substitution of Ni shifting the diffraction peaks of LaNiO_3_ to the left slightly. SEM imagery showed a coral‐like morphology with an average particle size of ~50 nm for the best performing Fe‐doped catalyst, around half the average size of the undoped LaNiO_3_. The catalyst with the lowest OER onset potential was LaNi_0.8_Fe_0.2_O_3_, with an overpotential of 391 mV at 10 mA cm^−2^ and Tafel slope of 102.8 mV dec^−1^; significantly higher values than those formerly reported for the same material by H. Wang [173], a trait that could be partially attributable to the different morphologies and thus different active surface areas of the materials. BET analysis of the coral‐like materials could provide a direct comparison of specific surface area between the catalysts from the two different studies, although a higher reported *C*_dl_ of the coral‐like materials would suggest a higher ECSA than that of the nanorods reported by H. Wang, indicating that surface area may not be the dominating factor in the differing performances. XPS analysis of the coral‐like catalyst indicated an increase in the Ni^3+^/Ni^2+^ ratio upon Fe doping, an opposing trend to that previously found by L. Wang et al. [205], along with a subsequent increase in oxygen vacancies, while it was also found that LNFO showed an increase in the Fe^3+^/Fe^2+^ ratio compared to LaFeO_3_. Therefore, while the exact reason remains elusive, it appears that a Ni:Fe ratio of 8:2 is optimal.

Yang et al. investigated the effect of Fe doping in La_0.5_Ba_0.5_CoO_3‐δ_ (LBC) perovskites, reporting that the optimal Fe‐doped material, La_0.5_Ba_0.5_Co_0.6_Fe_0.4_O_3‐δ_ (LBCF_0.4_), demonstrated a 42% reduction in overpotential compared to LBC (η_10_ of 301 vs. 473 mV) with a 36‐fold increase in turnover frequency at 300 mV [209]. Utilising a sol–gel synthesis approach via the EDTA/ citric acid complexing route, a range of La_0.5_Ba_0.5_Co_0.6_Fe_*x*_O_3‐δ_ (*x* = 0, 0.2, 0.3, 0.4, 0.5, 0.5, 1) materials were prepared and their surface chemistry probed with XPS and XAS. LBCF_0.4_ was found to have the highest Fe^4+^ content, while the Co—O bond was elongated compared to undoped LBC, with a reduced coordination number of oxygen around the Co atoms indicating increased oxygen vacancy concentration. These changes in the local structure have a significant role in modulating the catalytic activity of LBCF_0.4_: DFT calculations showed an upward shift of both the O 2p and Co 3d band centres in LBCF_0.4_ compared to LBC; after this shift, the energy difference between the Co 3d and O 2p band centres is reduced, which promotes the charge transfer from Co atoms to oxygen adsorbates. A similar perovskite, La_0.8_Ba_0.2_CoO_3_, was used by Mahmoudi et al. to investigate the effect of Fe doping [210]. The results largely corroborate the previously discussed findings of Yang et al. [209]: 30% substitution of Co by Fe was found to reduce the OER onset potential by 90 mV compared to the undoped material, while the mass activity improved from 7.14 to 17.36 A g^−1^.

Hu et al. reported a BiCoO_3_ perovskite prepared through solid‐state synthesis with a pyramidally coordinated TM centre (CoO_5_ rather than CoO_6_) that resulted in an ordered arrangement of oxygen vacancies and offered exceptional OER performance, with a η_500_ value of 391 mV and η_1000_ of just 402 mV [87]. This remarkable activity was attributed to several factors: the improved electrical conductivity due to the high oxygen vacancy concentration; increased access to active sites, again due to high oxygen vacancy concentration and to the pyramidal coordination of the TM; and the change in symmetry of the ligand field due to the pyramidally coordinated TM centre, which breaks the degeneracy of the $d_{z^{2}}$ and $d_{x^{2}-y^{2}}$ orbitals resulting in a split of the *e*_g_ group (Figure 12e). This leads to reduced hybridisation with the O 2p ligands, resulting in a decrease in energy of the $d_{x^{2}-y^{2}}$ orbital, due to elongation and angle distortion of the Co—O bonds, while electron transfer between the surface Co and adsorbed intermediates is directly promoted by the strong covalency between the Co and oxygen‐containing adsorbates enabled by the $d_{z^{2}}$ orbital. Most remarkably, when the Co in the BiCoO_3_ structure is partially replaced by Fe (denoted BCFO) the activity of the catalyst increases further, with a η_500_ value of 293 mV and η_1000_ of 303 mV; to the best of our knowledge, these are to date the lowest reported overpotentials at high current densities for a perovskite OER electrocatalyst.

### 6.3 Multi‐Metal Doping

**FIGURE 13:**
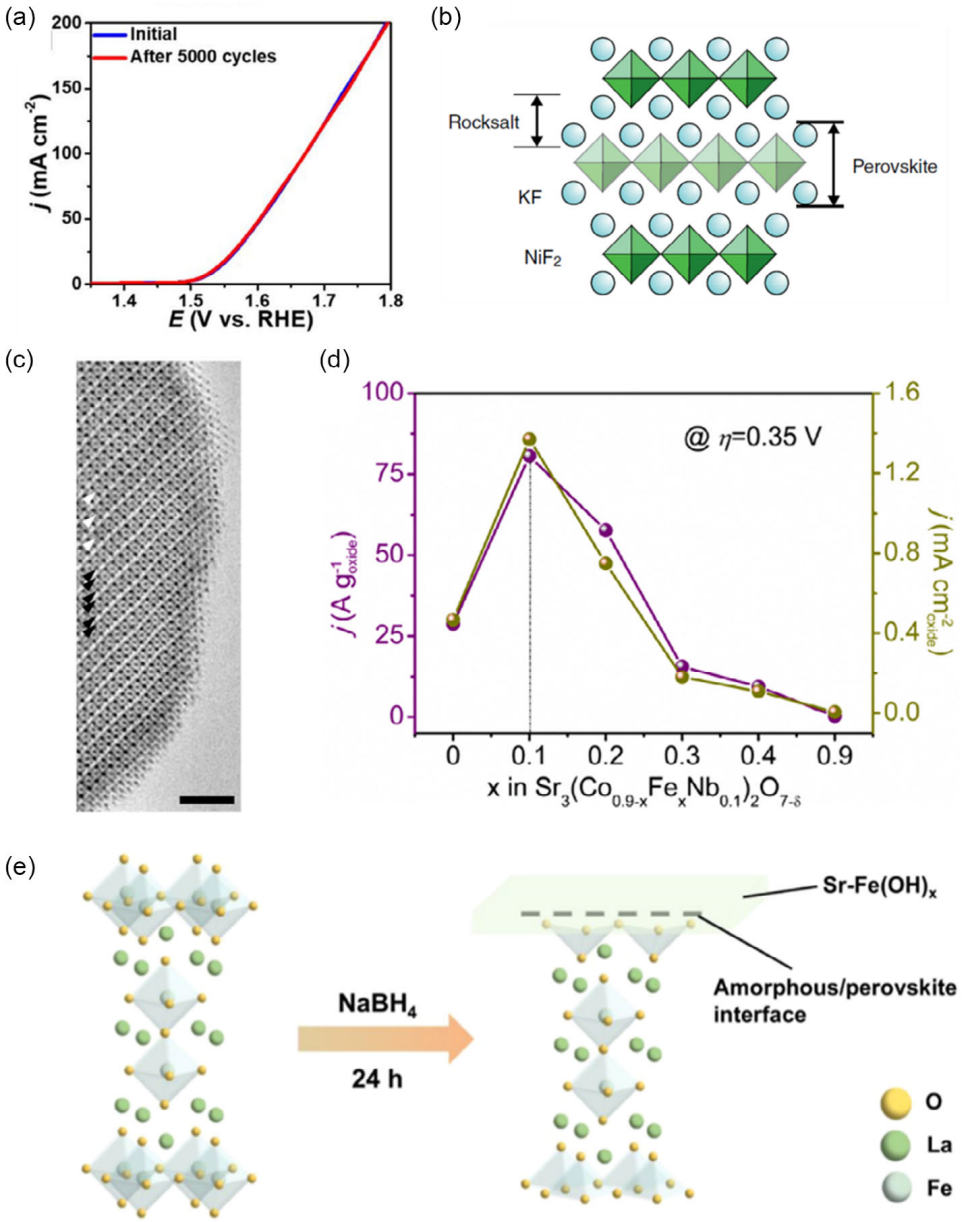
(a) LSV curves of LSPFCNO before and after 5000 cycles, reproduced with permission [213], Copyright 2025, Royal Society of Chemistry; (b) the ideal Ruddlesden–Popper structure of K_2_NiF_4_, reproduced with permission [114], Copyright 2016, Wiley; (c) ABF‐STEM of the RP oxide La_0.5_Sr_1.5_Ni_0.7_Fe_0.3_O_4±δ_, reproduced from [214] under the Creative Commons CC‐BY licence; (d) volcano plot showing different % of Fe substitution into RP structured Sr_3_(Co_0.9‐*x*_Fe_*x*_Nb_0.1_)_2_O_7‐δ_, reproduced from [215] under the Creative Commons CC‐BY licence; and (e) synthesis process of amorphous hydroxide/ perovskite interface, reproduced with permission [88], Copyright 2024, American Chemical Society.

Multi‐metal doping has been shown to be an effective method for improving the OER activity of a range of perovskites. Luo et al. successfully synthesised the hexagonal perovskite Ba_0.9_Sr_0.1_Co_0.8_Fe_0.1_Ir_0.1_O_3‐δ_ (BSCFI‐91) by sol–gel method and confirmed the structure using XRD and selected area electron diffraction (SAED) [211]. Based on the parent compound Ba_0.9_Sr_0.1_Co_0.8_Fe_0.2_O_3‐δ_ (BSCF‐91), it was found that substituting small amounts of Ir for Fe did not affect the hexagonal crystal structure. The prepared BSCFI‐91 catalyst exhibited improved OER activity compared to the parent BSCF‐91 catalyst with η_10_ = 300 mV, attributed to the reduction of Co valence, due to the slightly increased B–O distance caused by the large ionic radius of Ir^4+^; the O p‐band centre moving closer to the Fermi level; and optimal O_2_^2‐^/O^‐^ active sites on the catalyst surface. Of note is that the researchers attempted to dope the same Ir content into the B‐site of the cubic perovskite Ba_0.5_Sr_0.5_Co_0.8_Fe_0.2_O_3‐δ_ (BSCF‐55) to form Ba_0.5_Sr_0.5_Co_0.8_Fe_0.1_Ir_0.1_O_3‐δ_ (BSCFI‐55), but the prepared sample was found to be impure, suggesting that it is difficult for this compound to sustain a single, stable phase structure. Selvadurai et al. tailored the Fe cation sites of LaFeO_3_ through the simultaneous doping of Cr and Mo to give the LaFe_1‐*x*_Cr_*x*‐*y*_Mo_*y*_O_3‐δ_ (LFCMO) series of oxygen‐deficient perovskites [177]. The catalyst materials were produced via sol–gel synthesis, with a 2 h calcination in air at 900°C with a proportion subjected to a reduction in a 90:10 mixture of Ar/H_2_ at 900°C for 1 h. The samples that were reduced displayed better OER activity than the corresponding unreduced samples, as well as larger *C*_dl_ values, suggesting that the reduction process also resulted in more available active sites and thus a larger ECSA. It was found that the best performing catalyst was the LaFe_0.75_Cr_0.15_Mo_0.10_O_3‐δ_ sample that had been subjected to the reduction process (H‐LFCMO), which exhibited η_10_ value of 263 mV, compared to 320 mV for standard LFCMO, with a Tafel slope of 97 mV dec^−1^ as well as having the lowest charge transfer resistance of all the tested catalysts, found through electrochemical impedance spectroscopy (EIS). XPS analysis before and after OER testing showed a decrease in the Mo^6+^ peak intensity and an increase in peaks corresponding to Mo^4+^ and Mo^5+^ after testing, demonstrating the importance of Mo in the OER activity of the catalyst. Moreover, a decrease in the lattice oxygen peak after testing suggested that the OER could be proceeding, at least partially, through the LOM mechanism. Nguyen et al. also investigated the incorporation of Cr into the B‐site of a lanthanum‐based perovskite [191]; in this study, a co‐precipitation method was used to produce high entropy perovskite oxides (HEPOs) consisting of five consecutive first‐row TMs (Cr, Mn, Fe, Co and Ni) at the B‐site in both equimolar and non‐equimolar ratios. The best performing HEPO was found to be La(CrMnFeCo_2_Ni)O_3_ (designated L5M2Co), which exhibited an η_10_ of 325 mV with a Tafel slope of 51.2 mV dec^−1^ and good stability when tested over a 50 h period at a current density of 10 mA cm^−2^; La(CrMnFeCoNi_2_)O_3_ (L5M2Ni) had the second best performance, with η_10_ = 335 mV and a Tafel slope of 58.1 mV dec^−1^. XRD analysis showed that the HEPOs all presented a single‐phase orthorhombic structure, which was confirmed by TEM for L5M2Co, while infrared (IR) spectroscopy showed a broad band at 625 cm^−1^ corresponding to Jahn–Teller distortion in the MO_6_ octahedra, possibly arising from the existence of multiple TMs in different oxidation states. XPS analysis of L5M2Co found the valence ratios of Cr^6+^/Cr^3+^, Mn^4+^/Mn^3+^, Fe^3+^/Fe^2+^ and Co^3+^/Co^2+^ to be 0.82, 0.74, 0.71, and 2.81, respectively. Interestingly, only Ni^2+^ was found in the Ni 2p spectrum which contrasts with many Ni containing OER catalysts, where a quantity of Ni^3+^ is often found [80, 205]; only LaL5M2Ni showed the presence of the Ni^3+^ ion, with a Ni^3+^/Ni^2+^ ratio of 1.09, while elevated Mn^4+^/Mn^3+^ (1.55) and Fe^3+^/Fe^2+^ (1.31) ratios, when compared with the other HEPOs, were also observed. The turnover frequency of L5M2Co was the highest of all HEPOs (0.027 s^−1^) however, the calculated ECSA was amongst the lowest, indicating that the high catalytic performance is due to the electronic structure of the material rather than any morphological factors. It has been suggested that high oxidation states are more favourable to high catalytic activity [212], due to the higher likelihood of the acceptance of an electron from H_2_O, and all of the HEPOs (except for L5M2Ni) follow this trend, with the best performing L5M2Co containing the largest amounts of Cr^6+^, Mn^4+^, Fe^3+^ and Co^3+^ [191]; however, L5M2Ni contained the highest number of oxygen vacancies which could account for its relatively high activity. Using the same constituent elements, Verhage et al. synthesised an epitaxial film of a HEPO through PLD and examined the surface pre‐ and post‐OER testing using various scanning probe microscopy techniques [206]. The surface was found to undergo noticeable changes after OER testing, transitioning from a relatively flat morphology before testing to a rough surface after testing, while the OER current was found to initially decrease at the beginning of electrochemical testing before recovering and stabilising after approximately 15 cycles. This change in morphology coincided with a decrease in surface content of Mn, Cr, and Fe, which likely diffused into the electrolyte during OER cycling, while Ni and Co content persisted in significant amounts. Positive biasing of the probe tip was found to induce surface decomposition and degradation, leading to the postulation that the structural reorganisation was attributable to the leaching of oxygen anions, resulting in the accumulation of vacancies (Figure 12f). These vacancies subsequently act as charge‐trapping sites and influence bond strengths within the lattice, leading to the dissolution of elements into the electrolyte. Jiao et al. also synthesised a HEPO, with La, Pr, and Sr in the A‐site, and Fe, Co, and Ni in the B‐site [213]. Synthesised via the sol–gel method, the so called LPSFCNO showed good OER activity and excellent durability, with an η_10_ value of 304 mV that remained virtually unchanged after 5000 CV cycles (Figure 13a). The OER showed strong pH dependence, with oxidation current rising significantly as pH was increased, suggesting that LOM plays a dominant role in the OER mechanism. Surface amorphisation was detected as potentials were applied using in situ Raman spectroscopy, evidenced by the gradual reduction of the NiO_6_ vibrational mode band at 400 cm^−1^, which completely disappeared around 1.50 V; meanwhile, broad Raman bands around 1150 cm^−1^, attributable to the formation of active oxygen/superoxide species and another strong indicator of the LOM process, appeared above 1.50 V.

### 6.4 Ruddlesden–Popper Oxides

RP oxides, with the general formula A_*n* + 1_B_*n*_O_3*n* + 1_, are a layered series of perovskite‐type structure consisting of *n* consecutive layers of perovskite oxide A_*n*−1_B_*n*_O_3*n*−1_, one octahedron thick and cut parallel to the (100) planes, interlayered with a rock salt type structure with the formula AO [114, 216]. The ideal RP structure is exemplified by K_2_NiF_4_ (Figure 13b). RP structures have received considerable attention as OER catalysts due to their chemical flexibility, facile structural tunability and labile lattice oxygen that presents the opportunity for LOM participation [215, 216]. Forslund et al. reported a series of La_0.5_Sr_1.5_Ni_1‐*x*_Fe_*x*_O_4 ± δ_ RP oxides produced via a modified Pechini synthesis approach that displayed improved OER performance and stability compared with the corresponding basic perovskite structure [214]. XRD analysis produced a pattern characteristic of a first‐series (*n* = 1) RP crystal structure, corresponding to a body‐centred tetragonal unit cell with space group I4/mmm, which was confirmed through Rietveld refinement of the XRD data. Annular bright field scanning TEM (ABF‐STEM) showed perfect stacking of the perovskite and the rock salt layers without stacking faults or amorphisation (Figure 13c). It was found that 30% Fe substitution of Ni in this structure resulted in the highest catalytic activity, with mass activities of 1930 mA mg^−1^_oxide_ and 32.7 mA cm^−2^_oxide_ at 1.63 V (vs. RHE) and η_10_ = 360 mV with a Tafel slope of 44 mV dec^−1^. Meng et al. recently developed a ferromagnetic RP oxide La_2_CoO_4_, grown by PLD, and compared the performance to nonmagnetic LaCoO_3_, synthesised in the same way [217]. The OER activity of the RP oxide was around 2 orders of magnitude higher than LaCoO_3_, with a current density of 16.80 mA cm^−2^ recorded at 2.064 V (vs. RHE) for La_2_CoO_4_, compared to 0.24 ma cm^−2^ for LaCoO_3_. Interestingly, the more active RP La_2_CoO_4_ was found to have a lower proportion of oxygen vacancies (14.4%) compared to LaCoO_3_ (22.8%), suggesting that the AEM is the more favourable OER mechanism on La_2_CoO_4_, while a higher percentage of surface OH^−^ was found on La_2_CoO_4_ (33.7%) compared to LaCoO_3_ (22.9%) which could be favourable for OER activity via the AEM mechanism. Theoretical calculations were carried out in order to assess the role of magnetic order in the OER activity of La_2_CoO_4_ and suggested that the ferromagnetic ground state could facilitate spin‐polarised electron exchange between the RP oxide and the adsorbed oxygen species, thus boosting OER activity_._ [218] Research by Zhu et al. demonstrated the activation of the lattice‐oxygen in RP oxides for improved OER activity via the LOM reaction pathway [215]. The RP oxide Sr_3_(Co_0.8_Fe_0.1_Nb_0.1_)_2_O_7‐δ_ (RP‐SCFN) was produced via sol–gel synthesis and tested against the perovskite SrCo_0.8_Fe_0.1_Nb_0.1_O_3‐δ_ (P‐SCFN) and a benchmark RuO_2_ catalyst. RP‐SCFN displayed a smaller onset potential than both the P‐SCFN and RuO_2_ (~1.45 V vs. RHE) with a small η_10_ of 334 mV, a Tafel slope of 57 mV dec^−1^ and a mass activity of 80.7 A g^−1^ at η = 0.35 V. When tested in KOH solution of different pH values, the RP‐SCFN displayed much stronger pH dependence than P‐SCFN and RuO_2_, implying that the OER proceeds via LOM in RP‐SCFN. Soft XAS showed the coexistence of Co^3+^/Co^4+^ in RP‐SCFN as well as a higher average Co valence state (~ +3.44) relative to P‐SCFN; this is close to the optimal Co valence state of +3.4 in SrCoO_2.7_ reported previously by Mefford [115]; some Co^2+^ ions were detected in P‐SCFN, however they were not present in RP‐SCFN. The high Co valence state in RP‐SCFN means that the Co—O bond is highly covalent, while higher covalency in perovskite oxides has previously been linked to higher LOM participation [34], adding importance to the observation of the pH dependency of RP‐SCNF. XAS data showed no change in the surface Fe and Nb oxidation state between RP‐SCNF and P‐SCFN. Different amounts of Fe (0 ≤ *x* ≤ 1) were incorporated into the material Sr_3_(Co_0.9‐*x*_Fe_*x*_Nb_0.1_)_2_O_7‐δ_ and a volcano‐like trend was observed (Figure 13d), with the 10% Fe substitution of RP‐SCFN displaying the highest activity and thus sitting at the top of the volcano. DFT calculations were then carried out to further glean information on possible active sites and the reaction mechanism of RP‐SCFN. Both AEM and LOM were considered, with LOM having a significantly lower theoretical thermodynamic overpotential (0.22 V) than AEM (0.71 V), giving further evidence that OER proceeds via LOM on RP‐SCFN. Efforts to synthesise Nb‐free materials resulted in impure structures, suggesting that Nb^5+^ plays a key role in the stabilisation of the RP structure. It has been previously suggested that partial substitution of metal cations with another metal cation in a higher oxidation state (e.g. Nb^5+^, La^3+^) can result in the stabilisation of the perovskite phase structure, thus enhancing the long‐term durability of the catalyst [219]. This effect has also been previously noted specifically for RP structures with Nb^5+^ substitution [220].

### 6.5 Interface Engineering

Interface engineering is a promising strategy for regulating the electronic structure of materials, often resulting in lattice stress and charge transfer between two phases and subsequently leading to desirable properties such as improved ion transport and synergistic catalytic effects, while avoiding the disorder that is introduced by dopant atoms [158, 221, 222]. To this end, Paudel et al. first used DFT calculations to predict the band alignment and charge transfer across an LaFeO_3_/LaNiO_3_ heterointerface and found a valence band offset of ~0.2 eV between the LaNiO_3_ and LaFeO_3_ layers [222]. This valence band offset resulted in the nondegenerate p‐doping of the LaFeO_3_, with a high hole density induced from the charge transfer, although the authors note the difficulty in quantitative prediction of band offsets at complex oxide interfaces due several factors such as various magnetic states [222, 223]. When synthesised in the lab, the LaFeO_3_/LaNiO_3_ heterostructure showed up to a 275‐fold increase in OER activity (based on current density achieved at a potential of 1.6 V vs. RHE) compared to LaFeO_3_ alone, attributed to the small valence band offset which promotes electron transfer from the valence band of LaFeO_3_ to LaNiO_3_ by lowering energy barriers for charge carriers, verified by in situ XPS and the theoretical results obtained through DFT [222]. Zhao et al. also utilised DFT to investigate the interface in a biphasic composite structure of La_0.6_Sr_0.4_CoO_3_/MoS_2_, with calculations showing that the two‐phase interface modulated the bonding of reaction intermediates, while in situ Ramen spectroscopy identified cobalt (hydro)oxides with high oxidation states as surface reactive intermediates [224]. Recent work by Yan et al. investigated the interfacial electron transfer in an LaCoO_3_/Ti_3_C_2_T_*x*_ composite_._ [201] Titanium carbide MXene (Ti_3_C_2_T_*x*_) nanosheets were first prepared before combining with LaCoO_3_ using a urea‐assisted ball milling approach. When the LaCoO_3_ and Ti_3_C_2_T_*x*_ were mixed in a 1:1 ratio, uniform, granular particles of LaCoO_3_ were found to be wrapped by sheets of Ti_3_C_2_T_*x*_ just a few layers thick, maximising interfacial contact, while other ratios resulted in agglomeration of particles and a thicker Ti_3_C_2_T_*x*_ layer. This 1:1 composition yielded the best highest electrochemical activity, achieving a current density of 10 mA cm^−2^ at a potential of 1.65 V (vs. RHE) compared to 1.69 V for IrO_2_, while Ti_3_C_2_T_*x*_ tested on its own was unable to reach 10 mA cm^−2^ within the specified experimental range. The mass activity of the 1:1 composite (57 mA mg^−1^) was significantly higher than any of the other ratios tested (1:5, 1:2, 2:1, 5:1), which were all between 10 and 20 mA mg^−1^. The higher activity was of the 1:1 composite was attributed to the optimised interfacial contact between the LaCoO_3_ and Ti_3_C_2_T_*x*_ phases, which maximises interfacial charge transfer, while the polar terminal groups of the MXene phase enhance electrolyte access.

Han et al. synthesised an amorphous hydroxide/perovskite interface consisting of Sr‐Fe(OH)_*x*_ and the RP oxide Sr_3_Fe_2_O_7‐δ_ through a facile reduction strategy using a highly concentrated solution of NaBH_4_ (Figure 13e) [88]. The hydroxide/RP composite resulted in a significant drop in overpotential compared to the unmodified RP oxide, from 270 to 180 mV at 10 mA cm^−2^, with in situ Ramen spectroscopy indicating that the interface favours the adsorption of reaction intermediates, improves interfacial charge transfer, and facilitates the activation of the LOM pathway. When assembled in an AEMWE with a Pt/C cathode, a full‐cell potential of 2.32 V was achieved when operated at 1 A cm^−2^ at 50°C, while a current density of 300 mA cm^−2^ at the same temperature required a cell potential of just 1.58 V, with a degradation rate of 1.3 mV h^−1^. Lei et al. produced a perovskite (SrCoO_2.64_)‐ RP (PrSr_3_Co_1.5_Fe_1.5_O_10‐δ_) hybrid, labelled as AB0.8, with a heterointerface between the two component materials [61]. They found through extended X‐ray absorption fine structure (EXAFS) analysis that the structure was rich in oxygen defects, resulting in increased oxygen ion diffusion. Using temperature programmed reduction, it was elucidated that the high oxygen vacancy concentration in AB0.8 led to an activation of surface lattice oxygens, with LOM subsequently confirmed through ^18^O isotope‐labelling DEMS. The η_10_ of AB0.8 was 280 mV, considerably lower than either the separate perovskite (430 mV) and RP (394 mV) phases and also lower than a mixture of the two materials (353 mV).

### 6.6 Performance Comparison

As previously mentioned in Section 3.2, it is necessary to be able to make direct comparisons between both the activity and stability of different catalysts. In Table 2, we have collated some of the best performing recently reported materials from the aforementioned studies and present the commonly reported performance metrics of η_10_, Tafel slope, and stability at current density *j*. We reiterate here the urgent need to update and standardise testing procedures and performance metric reporting; many of the studies covered in this work report only a η_10_ value and CP stability testing at 10 mA cm^−2^, which tells little of a catalyst's ability to perform at high current density, as is standard in industrially operated electrolysers. Furthermore, to be able to make a proper comparison between the stability of different catalysts, we believe that the use of long‐term CA testing should become more commonplace, as here the electro‐oxidation conditions are constant, making a direct comparison between catalyst stability possible.

**TABLE 2:** The best performing recently reported perovskite OER electrocatalysts.

Perovskite	**η**_**10**_ **,** **mV**	**Tafel slope, mV dec** ^**−1**^	Stability	Electrolyte	**[Reference]** **(Year)**
Sr_3_Fe_2_O_7−δ_	180	48	200 h @ 300 mA cm^−2^	1 M KOH	[88] (2024)
La_0.9_CoFe nanotubes	244	59.1	20 h @ 10 mA cm^−2^	1 M KOH	[179] (2024)
Fe‐doped BiCoO_3_	256 η_1000_ = 303	44	40 h @ 10 mA cm^−2^	1 M KOH	[87] (2023)
Fe‐etched LaNiO_3_	280	48	300 h @ 10 mA cm^−2^	1 M KOH	[204] (2025)
(SrCoO _2.64_)‐ RP (PrSr_3_Co_1.5_Fe_1.5_O_10‐δ_) hybrid	280	55.9	120 h @ 10 mA cm^−2^	1 M KOH	[61] (2024)
Bi_0.15_Sr_0.85_Co_0.8_Fe_0.2_O_3−δ_	283	61.9	167.5 h @ 10 mA cm^−2^	0.1 M KOH	[137] (2023)
Ba_0.5_Sr_0.5_Co_0.8_Fe_0.2_O_3−δ_	303	47	100 h @ 200 mA cm^−2^	6 M KOH	[168] (2023)
(LaPrSr)(FeCoNi)O_3_	304	59.4	100 h @ 10 mA cm^−2^	1 M KOH	[213] (2025)
Sr_0.5_Ca_0.5_Co_0.5_Fe_0.5_O_3−δ_	310	55.9	100 h @ 10 mA cm^−2^	1 M KOH	[164] (2024)
LaCo_0.75_Fe_0.25_O_3_	310	58	100 h @ 10 mA cm^−2^	1 M KOH	[170] (2022)
La_0.8_Ba_0.2_Co_0.7_Fe_0.3_O_3_ ‐ Ag_0.1_	317	101	24 h @ 10 mA cm^−2^	0.6 M KOH	[210] (2025)
La(CrMnFeCo_2_Ni)O_3_	325	51.2	50 h @ 10 mA cm^−2^	1 M KOH	[191] (2021)
La_0.9_Ce_0.1_CoO_3_	343	64	12 h @ 10 mA cm^−2^	1 M NaOH	[202] (2025)

We highlight Han's work on amorphous hydroxide/perovskite interfaces [88] for achieving the lowest reported η_10_ value to date (180 mV), with excellent stability at 300 mA cm^−2^ for 200 h, which emphasises the impact of precisely engineered interfaces on catalyst performance and showcases why this strategy is emerging as one of the most promising approaches to rational design of catalyst materials. Meanwhile, Hu's work on pyramidally‐coordinated BiCoO_3_ [87] accomplished exceptional high current density performance, with a reported η_1000_ value of just 303 mV and good stability over 200 h (although different current densities were used across this timespan, so only stability at 10 mA cm^−2^ is reported in Table 2); this study demonstrates why it is sometimes important to ‘think outside of the box’ and trial catalyst chemistries that have no prior history in OER catalysis, rather than only relying on iterative improvement of pre‐established catalysts.

## 7 Conclusions and Future Directions

### 7.1 Outlook and Summary

The highly tuneable properties of perovskites make them desirable for use as catalysts for the OER, although it is clear that the performance of these materials is not yet where it needs to be for them to be considered as the cutting edge of OER catalysts. While encouraging progress has been made in improving the catalytic performance and stability of perovskite OER electrocatalysts, there is still a lot of work needed to further reduce the overpotential and improve stability at commercially relevant current densities.

Of paramount importance is establishing a standardised way of reporting results, so that data from different research groups is directly comparable. Although reporting overpotentials at 10 mA cm^−2^ is the most common practice, this tells us little about the performance of catalysts at a current density that is relevant for commercial applications. It is recommended that overpotentials are also reported at a minimum current density of 100 mA cm^−2^ moving forwards, while reporting at current densities of 500 and 1000 mA cm^−2^ is strongly encouraged. Furthermore, longer‐term stability testing for periods in the hundreds or thousands of hours, preferably in a full electrolyser cell testing setup rather than the more often used three‐electrode setup, should become more commonplace in order to progress perovskite OER electrocatalysts from out of the research laboratory setting and into commercial use.

It is clear that additional probing of the role that Fe plays in increasing OER activity is necessary, further insight of which would benefit not just research into perovskite OER catalysts, but also the wider field of TM‐based OER electrocatalysts, while also benefitting our understanding of the overall OER mechanism. In addition, continued investigation into the LOM and the effect that this mechanism has both on catalytic performance and catalyst stability would provide an excellent foundation on which to design the next generation of OER electrocatalysts.

### 7.2 Suggestions for Future Work

A key challenge for perovskite OER catalysts remains their long‐term stability under highly oxidising conditions. Many of the most active compositions undergo pronounced surface reconstruction, A‐site cation leaching, and amorphisation during operation, often forming a surface layer rich in B‐site metal (oxy)hydroxides. Rather than treating these processes solely as degradation pathways, future work should focus on compositions that undergo controlled, beneficial reconstruction, where a highly active surface phase is supported by a structurally robust perovskite bulk. Systematic studies that correlate A‐ and B‐site chemistry, oxygen non‐stoichiometry, and O 2p band position with reconstruction kinetics, A‐site dissolution, and loss of crystallinity would help to establish design rules for perovskites that maintain high activity while limiting irreversible mass loss over industrially relevant timescales.

The versatility of the perovskite structure and the ability to incorporate a vast number of different elements into the crystal lattice makes further research into high entropy perovskites and perovskite‐related phases, such as RP structures, a promising avenue for future research, with a near endless amount of different possible configurations. The sheer number of different possibilities could seem overwhelming; we believe that this presents an exciting opportunity to exploit the power of machine‐learning algorithms in order to quickly screen a large number of different configurations and converge on the most promising materials. A hurdle to this could be the often‐lengthy synthesis process required for perovskite materials; for the purposes of catalyst screening, we suggest the solid‐state synthesis approach as a suitable highthroughput method for producing novel materials and screening for electrocatalytic activity, before employing more refined processes such as sol–gel or hydrothermal to optimise characteristics such as morphology to further enhance performance.

To position perovskites as realistic choices for industrial application, direct benchmarking against state‐of‐the‐art TM‐based catalysts such as NiFe hydroxides is essential under identical testing conditions. Few studies rigorously compare perovskites head‐to‐head in terms of intrinsic performance (e.g., ECSA‐normalised and specific activity) and techno‐economic metrics (e.g., material cost, processing complexity, and lifetime). Future work should design comparative studies that quantify not only initial overpotentials at 10–1000 mA cm^−2^, but also degradation rates and failure modes. This will clarify where perovskites genuinely offer advantages over other materials.

## Author Contributions

**Mikey Jones**: conceptualisation (lead); data curation (lead); formal analysis (lead); investigation (lead); project administration (supporting); visualisation (lead); writing – original draft (lead); writing – review & editing (lead). **Adeline Loh**: writing – review & editing (supporting). **Cheng Lyu**: writing – review & editing (supporting). **Ida Nawrocka**: writing – review & editing (supporting). **Jack Corbin**: writing – review & editing (supporting). **Zhenyu Zhang**: writing – review & editing (supporting). **Xiaohong Li**: conceptualisation (equal); funding acquisition (lead); project administration (lead); supervision (lead); writing – review & editing (supporting).

## Funding

This work was supported by the Engineering and Physical Sciences Research Council Doctoral Training Partnership (EPSRC DTP), the EU's Horizon 2020 research and innovation programme under the MELODY project with grant agreement No. 875524, and the European Interreg 2 Seas Programme 2014–2020 co‐funded by the European Regional Development Fund under subsidy contract No [2S03‐019].

## Conflicts of Interest

The authors declare no conflicts of interest.

## Data Availability

The data that support the findings of this study are available in the supplementary material of this article.
